# Acceptance and Commitment Therapy for Psychosocial Outcomes in Children and Young People with Long-Term Physical Health Conditions: Systematic Review of Intervention Studies

**DOI:** 10.3390/children13050672

**Published:** 2026-05-12

**Authors:** Rachel Batchelor, Natasha Cogings, Christopher McCormack, Matthew Hotton

**Affiliations:** 1 The Oxford Institute of Clinical Psychology Training and Research, University of Oxford, Oxford OX3 7JX, UK; natasha.cogings@nottshc.nhs.uk (N.C.); christopher.mccormack@hmc.ox.ac.uk (C.M.); matthew.hotton@psy.ox.ac.uk (M.H.); 2 Oxford Health NHS Foundation Trust, Oxford OX4 4XN, UK; 3 Oxford University Hospitals NHS Foundation Trust, Oxford OX3 9DU, UK

**Keywords:** acceptance and commitment therapy, child and adolescent, long-term physical health conditions, paediatrics, psychosocial outcomes

## Abstract

**Highlights:**

**What are the main findings?**

- Preliminary evidence indicates potential benefits of acceptance and commitment therapy (ACT) for improving a range of psychosocial outcomes in children and young people (CYP) with long-term physical health conditions (LTCs), although findings regarding factors associated with these effects are mixed.
- Quantitative findings from a small number of studies suggest ACT interventions are acceptable to CYP with LTCs.

**What is the implication of the main finding?**

- ACT shows potential promise as a transdiagnostic intervention for CYP with LTCs, supporting its integration into paediatric psychosocial care pathways.
- High risk of bias, small sample sizes and limited follow-ups highlight the need for larger, rigorous trials with active controls, long-term assessment and diverse populations to strengthen evidence and guide clinical guidelines.

**Abstract:**

**Background/Objectives:** Children and young people (CYP) with long-term physical health conditions (LTCs) are at greater risk of psychosocial difficulties. Systematic reviews on adults with LTCs have supported acceptance and commitment therapy (ACT) in improving several psychosocial outcomes. This systematic review aimed to investigate the effectiveness of ACT for CYP-reported psychosocial outcomes among CYP with LTCs. It also examined the factors associated with the effects and the quantitative acceptability of the included ACT interventions. **Methods:** Eligible studies used a quantitative experimental design to evaluate ACT for CYP-reported psychosocial outcomes in CYP (≤18 years old) with LTCs. Only studies published in English in peer-reviewed journals, from any year, were included. CINAHL (EBSCO), Cochrane Library, Embase (Ovid), MEDLINE (Ovid) and PsycInfo (Ovid) were systematically searched. Google Scholar and Web of Science were also searched, and forward and backward citation searching was completed for included papers. Research quality was appraised using Cochrane risk-of-bias tools. Results were narratively synthesised. **Results:** Sixteen studies (nine randomised controlled trials (RCTs) and seven non-RCTs) from 19 reports met inclusion criteria, with 777 participants and five LTCs (chronic pain, diabetes, cancer, obesity and visual impairment). Findings provided preliminary support for the effectiveness of ACT on most CYP-reported psychosocial outcomes studied. Seven studies considered factors associated with intervention effects, with mixed findings. Acceptability was supported in the three studies that assessed it quantitatively. However, almost all studies had overall high/serious risk-of-bias ratings. **Conclusions:** There is preliminary evidence supporting potential benefits of ACT for improving psychosocial outcomes in CYP with LTCs, with limited but supportive findings for its acceptability. However, findings are constrained by high/serious risk of bias and small sample sizes. Larger, high-quality trials with active controls and longer follow-ups are needed to inform future care pathways. **Registration:** This systematic review was pre-registered (PROSPERO registration number: CRD42023425918).

## 1. Introduction

Over 1.7 million children and young people (CYP) have long-term physical health conditions (LTCs) in the UK [1]. CYP with LTCs are at greater risk of psychosocial difficulties, such as anxiety and depression [2,3]. Multiple factors may contribute, including illness symptoms, treatment side effects, uncertainty, reduced independence and identity/role changes [4].

Psychosocial difficulties in CYP with LTCs have been associated with adverse outcomes, including lower educational attainment, increased suicide risk and poorer illness management [5,6,7,8]. Consequently, health agencies have emphasised the need for psychological care in LTC populations [9,10].

### 1.1. Psychological Interventions

Psychological intervention research in CYP with LTCs has primarily focused on cognitive behavioural therapy (CBT) [11,12]. While CBT has the largest evidence base among CYP with LTCs, systematic reviews have reported mixed findings, with moderate–high risk of bias [11,12,13,14]. Such inconsistency in findings may partly reflect methodological limitations within the literature, including small and underpowered samples and substantial heterogeneity in study populations, intervention characteristics and study methods. Consequently, evidence remains insufficient to establish the effectiveness of CBT for some psychosocial outcomes, including health-related quality of life and depression, in CYP with LTCs [11], limiting firm conclusions regarding its overall effectiveness in this population.

Additionally, these mixed findings may reflect a broader mismatch between traditional CBT targets and the lived experience of LTCs. While CBT focuses on modifying maladaptive cognitions, some illness-related thoughts (e.g., uncertainty, symptom fluctuation and loss of control) may be realistic rather than distorted, limiting the applicability of cognitive restructuring in this context [15,16].

Therefore, interventions that promote adaptive responses to difficult internal experiences, flexible responding and engagement in meaningful activities while facing ongoing challenges may offer a more contextually appropriate therapeutic approach for CYP with LTCs.

### 1.2. Acceptance and Commitment Therapy

One such intervention is acceptance and commitment therapy (ACT) [17]. Based on relational frame theory, which describes how we construct meaning and understand the world by forming relationships between concepts [18], ACT uses a transdiagnostic approach that focuses on enhancing psychological flexibility. Psychological flexibility has been described as non-judgemental acceptance of thoughts, emotions and sensations; present-moment awareness; and taking meaningful values-based action, including during difficult experiences [19]. To target psychological flexibility, ACT incorporates six core processes (acceptance, cognitive defusion, committed action, present moment, self as context and values).

Psychological flexibility may be particularly relevant to LTC populations, as LTCs are persistent, making it important to maximise one’s fit with the situation rather than challenging it [20]. Systematic reviews on adults with LTCs have provided preliminary support for ACT on several psychosocial outcomes, including reducing anxiety, depression and psychological distress, and improving quality of life and symptom control [15,21,22,23,24], with clinical guidelines for chronic pain management in adults recommending ACT [25,26]. In contrast to CBT’s emphasis on cognitive change, ACT focuses on changing the relationship with thoughts, which may be particularly beneficial in LTC contexts, where distressing cognitions often reflect realistic health threats rather than cognitive distortions.

For CYP, ACT may provide a developmentally sensitive, adaptable treatment approach [27]. Developmentally, CYP tend to become more exploratory and seek independence [28,29], which ACT could facilitate through psychological flexibility. A recent meta-analysis of 14 randomised controlled trials (RCTs) showed ACT significantly improved anxiety and depression in clinical and non-clinical CYP [30]. Another meta-analysis of four RCTs comparing ACT and CBT for adolescents with anxiety found no significant differences in outcomes, despite CBT being the recommended intervention [31,32].

While there is promising support for ACT in adults with LTCs and CYP more broadly, and ACT is increasingly being studied in paediatric physical health settings, evidence has not yet been synthesised specifically for CYP with LTCs, despite this being a clinically distinct and growing population with complex psychosocial needs. Existing reviews either focus on adult LTC populations or mixed CYP samples [15,30], limiting conclusions for CYP with LTCs specifically. This represents an important gap, given the psychosocial difficulties in this population and the need for evidence-based tailored psychological interventions.

Importantly, this review focuses specifically on CYP-reported psychosocial outcomes. Psychosocial constructs are inherently subjective experiences, and discrepancies have been consistently observed between CYP self-reports and proxy (e.g., parent) reports of psychosocial outcomes [33,34,35]. Prioritising CYP-reported outcomes may provide a more valid understanding of whether ACT improves psychosocial difficulties from the perspective of the CYP receiving the intervention. As ACT is a transdiagnostic rather than disorder-specific approach, understanding factors associated with its effects, such as the processes or mechanisms of change through which ACT exerts its effects, is also important for improving its application [36].

### 1.3. Aims and Research Questions

This systematic review aims to evaluate the effectiveness of ACT on CYP-reported psychosocial outcomes among CYP with LTCs. It also aims to examine the factors associated with the effects and quantitative acceptability of the included ACT interventions.

#### 1.3.1. Primary Research Question

1. What is the effectiveness of ACT interventions for CYP-reported psychosocial outcomes in CYP with LTCs?

#### 1.3.2. Secondary Research Questions

2. What factors are associated with the intervention effects of the evaluated ACT interventions in CYP with LTCs?
3. What is the quantitatively rated acceptability of the evaluated ACT interventions in CYP with LTCs?

## 2. Materials and Methods

### 2.1. Protocol

The protocol for this systematic review was pre-registered on the international database of Prospective Register of Systematic Reviews (PROSPERO) in May 2023 (registration number: CRD42023425918). The Preferred Reporting Items for Systematic Review and Meta-Analysis (PRISMA) 2020 guidelines [37] were followed (see Supplementary File S1 for checklist).

### 2.2. Search Strategy

A systematic search of databases from inception was conducted on 20 August 2024, and updated on 3 February 2025. Searching of titles, abstracts and keywords was undertaken across the following databases: CINAHL (EBSCO), Cochrane Library, Embase (Ovid), MEDLINE (Ovid) and PsycInfo (Ovid). Medical Subject Headings (MeSH) were applied where appropriate, specifically in MEDLINE and the Cochrane Library. Google Scholar and Web of Science were also searched for additional papers. Lastly, forward and backward citation searches were completed for included papers.

### 2.3. Search Terms

The search strategy captured two main concepts: (i) CYP and (ii) ACT. LTCs were not included as a separate search concept in order to reduce the risk of excluding relevant studies due to variation in condition terminology and indexing across paediatric health populations. This broad approach aimed to capture ACT studies involving CYP with physical health conditions across a range of diagnoses. This approach aligns with a previous systematic review evaluating ACT in adults with LTCs, which similarly adopted broad search terms to maximise sensitivity across diverse LTCs [15].

The search strategy comprised the following terms: (Child* OR Adolescen* OR Teen* OR youth* OR young* OR juvenile* OR paediatric* OR pediatric* OR Boy* OR Girl* OR Schoolchild* OR Minor* OR “Under 18”) AND (“acceptance and commitment therapy” OR “DNA-V” OR “Discoverer Noticer Advisor?Values” OR “psychological flexibility”). The full, line-by-line search strategy for each database is provided in Supplementary File S2.

### 2.4. Inclusion and Exclusion Criteria

The PICO (Patient, Intervention, Comparison and Outcome) framework [38] was used to define the inclusion criteria (Table 1).

Study exclusion criteria included not in English, not peer-reviewed, qualitative-only studies and case studies.

### 2.5. Study Selection

All citations identified through the search were imported into Excel. After removing duplicates, one researcher (RB) screened all titles and abstracts. A second researcher (NC) independently screened a random 20%. The two reviewers had almost perfect agreement for title/abstract screening (*κ* = 0.88). RB then completed full-text screening for all remaining reports to determine eligibility, and NC conducted an independent screen of a random 25%. The two reviewers had almost perfect agreement for included/excluded studies for full-text screening (*κ* = 0.91). Two additional researchers (CM and MH) then conducted independent checks of all included full texts. At each stage, any discrepancies were resolved through discussion within the review team until consensus was reached, with re-examination of the full-text articles where necessary to ensure consistency and accuracy of inclusion decisions.

### 2.6. Data Extraction and Synthesis

Data were extracted using Cochrane’s data collection form for intervention reviews [40], modified for the current systematic review (see Supplementary File S3). RB extracted data from all included reports, with NC independently extracting a random 50% to ensure accuracy and reliability. The two reviewers had almost perfect agreement for data extraction (*κ* = 0.94). Discrepancies were discussed and resolved within the review team. Additional information was sought by contacting study authors.

Given the clinical and methodological heterogeneity across reports (e.g., study designs, LTCs, ACT interventions and outcomes), data were not sufficiently homogenous for quantitative pooling (i.e., meta-analysis). Therefore, results were narratively synthesised, following the Synthesis without Meta-Analysis (SWiM) guideline [41] (see Supplementary File S1 for checklist). Study characteristics (design and participants), intervention characteristics and CYP-reported psychosocial outcomes were reported in tabular form and narratively. Where available, factors associated with the effects of ACT and quantitative acceptability were also reported. Although the pre-registration for this systematic review specified examining mediators of ACT effects, the scope was broadened to include any analyses of factors associated with CYP-reported psychosocial outcomes, due to the limited number of studies reporting mediators.

Study outcome syntheses were organised by LTCs to account for population heterogeneity and enhance LTC-specific interpretability for research and practice. All time points and analyses were reported. In the absence of meta-analyses, potential reporting biases were described qualitatively. Regarding standardised metrics, where available, *p*-values and effect sizes for within-group and between-group analyses were extracted. Originally reported effect sizes were used due to the heterogeneity between studies in reported effect sizes. Effect size interpretations followed Cohen’s guidelines [42]. If not reported, appropriate effect sizes were calculated if data allowed. Inconsistent findings were qualitatively discussed regarding potential sources of heterogeneity.

### 2.7. Quality Appraisal Strategy

The Cochrane Risk of Bias 2 (RoB 2) [43] tool was used to assess the quality of RCTs. The Cochrane Risk of Bias in Non-Randomised Studies of Interventions (ROBINS-I) [44] tool was used to assess the quality of non-RCTs. RoB 2 and ROBINS-I assess five and seven domains, respectively, with each study’s overall risk of bias reflecting its highest domain rating [43,44]. Studies were not excluded from the synthesis based on risk of bias in order to retain the full scope of relevant evidence, following Cochrane guidance [45]. RB assessed the quality of all included studies, with NC independently appraising a random 30%. The two reviewers had perfect agreement (*κ* = 1) for overall risk of bias and almost-perfect agreement (*κ* = 0.82) at the domain level. Discrepancies were discussed within the review team until consensus was reached.

## 3. Results

**Figure 1:**
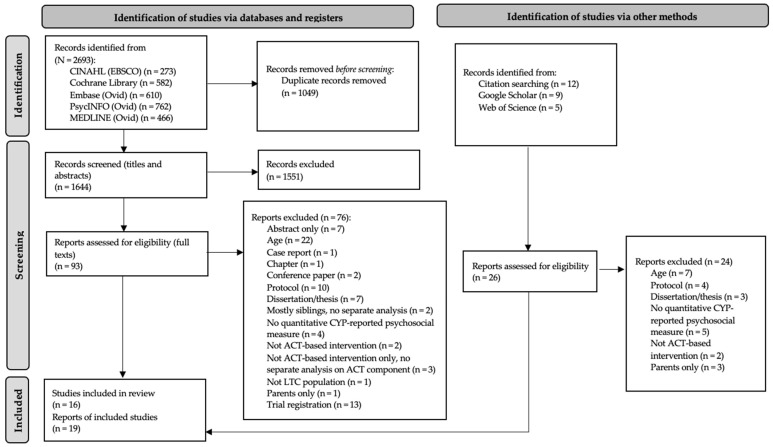
Preferred Reporting Items for Systematic Reviews and Meta-Analysis (PRISMA) flow diagram of selection process [37].

Overall, the database searches yielded 2693 citations. Citations were screened for duplicates; 1049 were removed. Titles and abstracts of the remaining 1644 articles were screened, and 1551 were excluded. The full texts of the remaining 93 articles were read and assessed for eligibility; 76 were excluded (see Figure 1 for reasons) and 17 were included. An additional 26 reports were identified through hand-searching Google Scholar and Web of Science and forward and backward citation searching of included reports; 24 were excluded and two were included. Overall, 16 studies from 19 reports were included. A PRISMA flow diagram [37] of this process is provided in Figure 1.

### 3.1. Study Designs and Participant Characteristics

Study designs and participants characteristics are presented in Table 2. Nine studies were RCTs [46,47,48,49,50,51,52,53,54]. Two RCTs [47,48] had secondary analyses in additional reports [55,56]. Three RCTs used active controls (psychoeducation, a meeting without offering solutions and make-up empowering sessions) [51,53,54], three used treatment-as-usual (TAU) [48,52] and two offered no intervention (NI) [49,50]. One RCT compared group ACT and individual ACT [46]. The remaining seven studies were non-RCTs, one with a control group (NI) [57] and six without a control group [58,59,60,61,62,63]. One non-RCT [61] had one additional report on secondary analyses [64].

Six studies were conducted in Iran [49,50,51,53,54,57]; four in Sweden [46,47,48,61]; two in Finland [48,60]; and one each in England [59], Italy [52], Romania [62] and the USA [63]. Five LTCs were included: chronic pain (six studies) [46,47,58,59,60,61], diabetes (four) [48,49,50,62], obesity (three) [51,52,63], cancer (two) [53,57] and visual impairment (one) [54].

Overall, 777 CYP were included, with samples of seven to 187. Ages ranged from 7 to 18 years old. Across the 14 studies that reported gender, all but two [49,50] had all or mostly females [46,47,48,51,52,54,58,59,60,61,62,63]. Only one study reported ethnicity, although not for all CYP [63].

### 3.2. Acceptance and Commitment Therapy Intervention Characteristics

ACT intervention characteristics are summarised in Table 3. Fourteen studies reported intervention contents [46,48,49,50,51,52,54,55,58,59,60,61,62,63], all covering at least four core psychological flexibility processes. While not explicitly mentioned, some processes could be inferred from the material described. However, levels of detail on intervention content varied across studies, and some processes may not have been reported and were therefore missed during extraction. Given limited reporting, the core psychological flexibility processes could not be identified for the cancer studies [53,57].

Nine studies reported parental involvement [46,47,48,51,58,59,60,61,63], including all six chronic pain studies. Four studies involved parallel or separate parent sessions/modules only [47,60,61,63]; three studies involved a combination of joint and separate CYP and parent sessions [46,58,59]; one study involved initial joint sessions for CYP and parents [51]; and in one study, parents only joined some of the first and last session [48].

Nine studies reported the delivery settings, with eight in medical settings [46,48,52,57,59,60,62,63] and one in an educational setting [63]. Fourteen studies reported ACT facilitator(s): eight by psychologists [48,49,50,51,52,58,61,62], three by a multidisciplinary team [46,59,60], one by a psychologist and dietician [63], one by a psychologist and physician 47 and one by a counsellor [53].

Interventions mostly included weekly sessions, totalling between three and 90 h of ACT-based support. Ten studies delivered ACT in groups (two-seven CYP) [48,49,50,51,53,54,57,58,60,63], four individually [47,52,61,62], one used a combined both group and individual format (i.e., participants received both) [59] and one compared group and individual delivery [46]. Fourteen studies delivered ACT face-to-face [46,47,48,49,50,51,52,53,54,57,58,59,60,63], one online [61] and one a mix (most online and some face-to-face) [62].

### 3.3. Quality Appraisal

**Figure 2:**
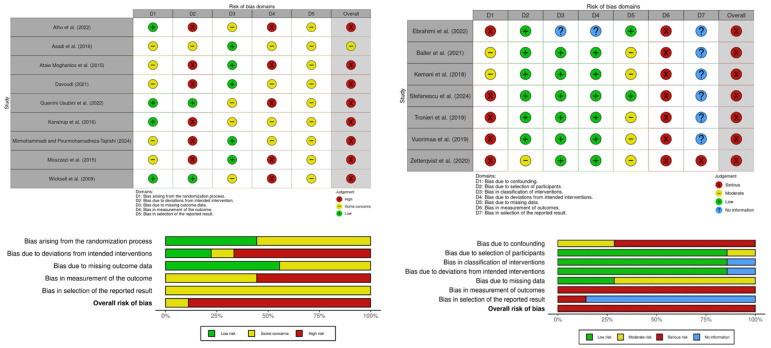
Quality-appraisal summaries. Note. Traffic-light plot and summary plot for randomised controlled trials, assessed using the Cochrane Risk of Bias 2 (RoB 2) [43] on the left (studies listed alphabetically). Traffic-light plot and summary plot for non-randomised controlled trials, assessed using the Cochrane Risk of Bias in Non-Randomised Studies of Interventions (ROBINS-I) [44] on the right (study with a control group listed first, followed by studies without a control group, listed alphabetically).

Results from the quality assessment are presented in Figure 2, created using the robvis app (version 0.3.0; University of Bristol, Bristol, UK) [65]. Following RoB-2 and ROBINS-I guidelines [43,44], quality was assessed for each outcome of interest (i.e., CYP-reported psychosocial outcomes). As ratings were identical across outcomes within each study, a single set of combined outcome ratings are presented per study. The effect of interest was assignment to intervention. The quality appraisal is summarised below (see Supplementary File S4 for a more detailed appraisal per domain).

#### 3.3.1. RCTs

Overall risk of bias was rated “high” for eight RCTs [46,47,48,49,50,51,52,54] and “some concerns” for one RCT [53]. However, this latter rating should be viewed with caution, as it reflects insufficient reporting in several domains, which, per tool guidelines, should be rated as “some concerns” rather than “no information” [43].

A strength of several studies was randomisation, with appropriate sequence generation and no substantial baseline imbalance [46,47,48,52]. Five studies reported insufficient information about randomisation [49,50,51,53,54]. Regarding missing outcome data, apart from one study which indicated no dropout [53], dropout rates varied between 7.14% and 37.5% from randomisation to final time point. Five studies reported dropout reasons: two reported non-health-related reasons [51,54], two reported ≤5% health-related dropouts [49,50] and one reported >20% health-related dropouts, with rates differing between groups [46]. In studies lacking reasons, dropout rates were similar across groups [47,48,52].

While all studies had appropriate outcome measurements, comparable between groups, all outcomes were self-reported (as required for inclusion eligibility). As participants were the outcome assessors and most studies used TAU or NI as controls [47,48,49,50,52], outcomes may have been influenced by awareness of the intervention received.

Across studies, no deviations from intended interventions were indicated; however, one study provided no information regarding intervention content [53]. However, all but two studies, one which indicated no dropout [53] and one which used intention-to-treat analyses [47], did not include all randomised participants in analyses. Although three studies were pre-registered [48,51,52], none included pre-specified analysis plans. Therefore, for all studies, there was insufficient evidence to assess selective reporting.

#### 3.3.2. Non-RCTs

Overall risk of bias was rated as “serious” for non-RCTs, indicating “important problems” [44]. However, without the “measurement of outcome” domain, which was rated as serious for all studies due to self-reported psychosocial measures being required for inclusion eligibility, two studies would be rated as “moderate” [58,59], indicating sound evidence for non-RCTs, though not comparable to well-performed RCTs [44].

A strength across studies was participant selection. Participant selection occurred before the intervention had begun and time points coincided for most participants; however, in one study, four-month follow-ups were completed by participants between 17 and 25 weeks post-ACT [61]. For most studies, intervention groups were also well-defined, with no deviations from intended interventions indicated.

Considering study weaknesses, all outcome measures were self-reported and subjective, with assessors therefore unblinded to the intervention received. Another common area of weakness was potential confounding. Although all studies excluded the risks of some confounders through eligibility criteria, only two studies controlled for potential confounders in analyses [58,59].

Missing data varied across studies. Two studies reported no or <5% dropouts or missing outcome data [57,62]. In the remaining studies, rates of dropout and missing data ranged from 14.3% to 42.8%. No reasons for attrition were reported, so the potential bias could not be ruled out. Regarding selection of the reported results, one study’s pre-registration specified follow-ups at four months and one year but collected four-month data inconsistently (17–25 weeks) and omitted the one-year data [61], indicating potential selective reporting. The remaining studies lacked pre-registration/a priori analysis plans, limiting assessment of reporting bias.

### 3.4. Study Outcomes

Each study reported findings of ACT intervention effects on between one and thirteen CYP-reported psychosocial outcomes. Seven studies reported findings on factors associated with ACT effects [48,52,55,58,59,60,64], and three studies reported quantitative acceptability data [54,56,63]. Three studies also reported qualitative acceptability data, which are beyond the scope of this systematic review [56,61,62].

Eight studies assessed outcomes at two time points (pre-intervention and post-intervention) [48,49,50,52,57,58,62,63], with another study adding an additional mid-intervention time point [46]. Seven studies included follow-ups; six had three time points, five with pre-intervention, post-intervention and one follow-up [51,53,54,59,61], and one with pre-intervention and two follow-ups from pre-intervention [60]; and one had four time points (pre-intervention, post-intervention and two follow-ups) [47]. Follow-ups ranged from 1 to 12 months post-intervention.

Study outcome data are narratively synthesised for each LTC below regarding (i) ACT intervention effectiveness, (ii) factors associated with the effects of ACT and (iii) acceptability of ACT. Notably, all included studies were rated as high/serious risk of bias, except one cancer study [53], rated as “some concerns” due to insufficient methodological reporting. Across studies, only one in chronic pain indicated potential selective reporting [61]; however, the absence of a priori analysis plans for the remaining studies limited assessments of selective reporting, as detailed in the quality appraisal.

#### 3.4.1. Chronic Pain

##### Effectiveness on CYP-Reported Psychosocial Outcomes in CYP with Chronic Pain

Outcome data from the chronic pain studies are presented in Table 4. Pain-related outcomes were assessed across studies. In two RCTs [46,47] and two non-RCTs [58,59,61] per outcome, significant improvements in pain intensity and pain interference were observed over time for CYP receiving ACT. Wicksell et al.’s [47] RCT found significantly greater improvements in ACT than TAU. In Kanstrup et al.’s [46] RCT, no significant between-group (group and individual ACT) differences were found.

Several other pain-related outcomes were assessed by one study each. Wicksell et al.’s [47] RCT reported significant improvements in pain-impairment beliefs and pain-related discomfort over time (to two-month follow-up) in ACT, with large effect sizes. Significant between-group differences were also in support of ACT (large effect sizes). In Kanstrup et al.’s [46] RCT, significant pre–post improvements in pain reactivity were found for both formats combined, with medium effect sizes, and no between-group (group and individual ACT) differences were found. Kemani et al.’s [59] non-RCT found pain-specific anxiety significantly improved pre–post-ACT.

Five studies assessed depression [46,47,58,59,61]. Non-RCTs [58,59,61] consistently found significant improvements in depression over time among CYP receiving ACT, with medium effect sizes reported by two studies [59,61]. Similarly, Kanstrup et al.’s [46] RCT found significant pre–post improvements in depression for both formats combined (medium effect size), with no significant between-group differences. However, Wicksell et al.’s [47] RCT found no significant within-group or between-group effects of ACT on depression. Notably, only Wicksell et al. [47] compared ACT to a non-ACT control.

For insomnia, Zetterqvist et al. [61] found significant improvements pre-ACT to follow-up (medium effect size), whereas Balter et al. [58] found no significant changes pre–post-ACT. Although both non-RCTs had overall ratings of serious risk of bias, methodological differences, such as Zetterqvist et al.’s [61] inclusion of a follow-up and potential selective reporting and Balter et al.’s [58] controlling for potential confounders, may, at least partly, account for such differences.

Three studies consistently supported the effects of ACT on psychological flexibility [46,58,61], with two studies reporting large effect sizes [46,61]. In Kanstrup et al.’s [46] RCT, no significant between-group (group and individual ACT) differences were found. Acceptance also significantly improved pre–post-ACT in one study [59], with a medium effect size.

Mixed findings were reported for outcomes of functioning. Wicksell et al.’s [47] RCT found significant improvements in functional disability over time in ACT (large effect size), but no between-group differences. Non-RCTs found significant pre–post-ACT improvements for social functioning and physical functioning [58,59], with small effect sizes reported in one [59]. Significant improvements over time (to three-month follow-up) in family functioning were reported in one non-RCT [59] (medium effect size), and significant pre–post improvements in emotional functioning were reported in another [58]. However, two non-RCTs found no effects on school functioning [58,61].

Of the remaining outcomes, Wicksell et al.’s [47] RCT found that kinesiophobia, mental quality of life and physical quality of life significantly improved over time in ACT (large effect sizes). Between-group differences were also significant for kinesiophobia and mental quality of life at post-intervention in support of ACT, with large effect sizes, but not for physical quality of life. No significant within- or between-group effects were found for internalising/catastrophising. Kemani et al.’s [59] non-RCT found no significant effects of ACT on development.

##### Factors Associated with CYP-Reported Psychosocial Outcomes in CYP with Chronic Pain

Regarding pain-related factors, in Vuorimaa et al.’s [60] non-RCT, higher pain risk profiles moderated the effectiveness of ACT, with the high-risk group reporting significant improvements over time (to 12 months from pre-intervention) in depression, pain frequency and pain catastrophising, but the medium-risk group reporting no significant changes. Furthermore, in Wicksell et al.’s RCT [47,55], post-ACT pain-impairment beliefs mediated changes in depression at both the one- and two-month follow-ups and in pain interference at the one-month follow-up only, while pain reactivity mediated changes in both depression and pain interference at both follow-ups. Other post-ACT outcomes (internalising/catastrophising, kinesiophobia, pain intensity or self-efficacy) were not significant mediators.

Changes in psychological flexibility were significantly positively associated with changes in adolescent pain acceptance in Kemani et al.’s non-RCT [59]. In Zetterqvist et al.’s non-RCT [61,64], higher acceptance in the early treatment phase was significantly related to larger decreases in pain interference (medium effect size), whereas defusion, values formation and committed action showed no significant effects.

Regarding neurodevelopment, Balter et al. [58] found that CYP with higher autistic traits or clinically significant autism/ADHD symptoms showed significantly greater pre–post improvements in insomnia and emotional functioning, but not in other outcomes (depression; pain intensity; pain interference; psychological inflexibility; or physical, school or social functioning).

##### Acceptability of Interventions in CYP with Chronic Pain

Not quantitatively studied.

##### Summary for CYP with Chronic Pain

Evidence from chronic pain studies suggests that ACT is associated with improvements in several CYP-reported psychosocial outcomes, though findings are somewhat mixed, and all studies were rated as having high/serious risk of bias. Pain intensity and pain interference consistently improved over time across two RCTs and two non-RCTs per outcome, with some evidence supporting ACT over TAU. Improvements in other pain-related outcomes were indicated by one study each. Psychological flexibility also showed consistent gains in two non-RCTs and one RCT, and improvements in acceptance were indicated by one non-RCT. Depression findings were less consistent: three non-RCTs and one RCT reported improvements, while another RCT with a non-ACT control found no significant effects. Notably, only the latter had a non-ACT comparator. Results for insomnia and functioning (e.g., social, physical, family and school) were variable, with some improvements but no clear pattern. These inconsistencies may be partly explained by methodological differences across studies, including the presence or absence of follow-up assessments, potential selective reporting and whether analyses accounted for confounding variables. Additional benefits were observed for outcomes such as kinesiophobia and mental quality of life; however, not all domains improved.

In terms of factors associated with CYP-reported psychosocial outcomes, evidence from an RCT suggests that pain-related factors (e.g., pain-impairment beliefs and pain reactivity) may mediate improvements in depression and pain interference, while a non-RCT indicated higher baseline pain risk profiles may moderate treatment effectiveness. Psychological flexibility was associated with pain acceptance in one non-RCT, and early treatment gains in acceptance were linked to reductions in pain interference in another non-RCT. One non-RCT also indicated that neurodevelopmental characteristics (e.g., autistic traits and ADHD symptoms) influence responsiveness to ACT for certain outcomes. However, no quantitative data on intervention acceptability were reported.

#### 3.4.2. Diabetes

##### Effectiveness on CYP-Reported Psychosocial Outcomes in CYP with Diabetes

Outcome data from the diabetes studies are presented in Table 5. Three outcomes, depression, psychological flexibility and stress, were assessed by two studies each. The effectiveness of ACT on stress outcomes was supported by both Moazzezi et al.’s [49] RCT, which found perceived stress (and its subscales) significantly improved pre–post-ACT compared with NI, and Stefanescu et al.’s [62] non-RCT without a control group, which found significant pre–post-ACT improvements in stress, all with large effect sizes. The effectiveness of ACT on psychological flexibility was also consistently supported, with Alho et al.’s [48] RCT supporting the effectiveness of ACT on diabetes-related psychological flexibility pre–post-ACT compared with TAU [48] (small effect size) and Stefanescu et al.’s [62] non-RCT reporting pre–post-ACT improvements in psychological flexibility (large effect size).

Although pre–post improvements in depression were supported in Ataie Moghanloo et al.’s [50] RCT compared with NI, Alho et al.’s [48] RCT found no significant between-group differences. However, the RCTs differed methodologically. Alho et al. [48] utilised a TAU control, while Ataie Moghanloo et al. [50] used NI. Unlike Alho et al. [48], Ataie Moghanloo et al. [50] also reported insufficient information on randomisation and lacked pre-registration.

The remaining outcomes were assessed by one study each. In Alho et al.’s [48] RCT, between-group analyses supported ACT for improving anxiety compared with TAU (small effect size). However, no significant between-group differences were found for diabetes-related quality of life and general quality of life. Between-group analyses in the RCTs comparing ACT and NI [49,50] supported the effectiveness of ACT on depression, feeling of guilt, psychological wellbeing and special health self-efficacy, all with large effect sizes. Significant improvements pre–post-ACT in patient–doctor relationship with a large effect were also found in Stefanescu et al.’s [62] non-RCT.

Notably, no ACT studies for CYP with diabetes included follow-ups, meaning long-term effectiveness could not be considered.

##### Factors Associated with CYP-Reported Psychosocial Outcomes in CYP with Diabetes

Diabetes-related psychological flexibility did not mediate the effects of ACT on anxiety in Alho et al.’s [48] RCT.

##### Acceptability of Interventions in CYP with Diabetes

In Alho et al.’s [48,56] RCT, most adolescents reported satisfaction with the ACT intervention including its length. All adolescents perceived benefits, and all but one reported that they would recommend the intervention to other adolescents.

##### Summary for CYP with Diabetes

Evidence from diabetes studies suggests that ACT shows promising, though not entirely consistent, benefits for CYP-reported psychosocial outcomes. Improvements in stress and psychological flexibility were consistently supported across studies, with both RCT and non-RCT designs reporting significant pre–post gains, often with large effect sizes. Findings for depression were mixed, with one RCT showing significant improvements compared to no intervention, while another RCT found no differences compared to treatment as usual, likely reflecting methodological differences. Additional outcomes showed some promising effects, including improvements in anxiety, psychological wellbeing, self-efficacy, guilt and patient–doctor relationships; however, quality-of-life outcomes did not significantly improve. However, all studies were rated as high/serious risk of bias, and no studies included follow-up assessments, limiting conclusions about long-term effectiveness. Psychological flexibility did not mediate anxiety outcomes in one RCT. Acceptability assessed by one RCT suggested high acceptability, with most adolescents reporting satisfaction, perceived benefits and willingness to recommend ACT.

#### 3.4.3. Obesity

##### Effectiveness on CYP-Reported Psychosocial Outcomes in CYP with Obesity

Outcome data from obesity studies are presented in Table 6. Three outcomes, depression, avoidance and fusion and stress, were assessed by two studies. Results for stress outcomes were consistent, with two studies finding no significant effects [52,63]. However, findings for depression and experiential avoidance and fusion were inconsistent. In Guerrini et al.’s [52] RCT comparing ACT+TAU, no significant effects for depression or experiential avoidance and fusion were found, yet in Tronieri et al.’s [63] non-RCT, depression scores decreased pre–post-ACT (large effect size) and increased for experiential avoidance (small effect size). However, Tronieri et al.’s [63] study lacked a control group and, as a feasibility study, had a considerably smaller sample size and no tests of statistical significance. Although both studies used the same measure for experiential avoidance and cognitive fusion, with Tronieri et al. [63] shortening the name, they employed different outcome measures for depression. Such methodological differences may account for the inconsistent findings.

The remaining outcomes were measured by one study each. The effectiveness of ACT on food addiction over time (to 12-week follow-up) was supported in between-group analyses of an RCT comparing ACT and psychoeducation on individual advice by Davoudi [51], although effect sizes could not be calculated.

In Guerrini et al.’s [52] RCT, the remaining findings were mixed. Emotional eating significantly improved pre–post-intervention in both ACT+TAU and TAU (effect sizes could not be calculated), although no significant between-group differences were found. However, anxiety levels increased in both groups (effect sizes could not be calculated), with a significantly greater increase in ACT+TAU over time. No significant effects for emotional dysregulation and psychological wellbeing were found. In Tronieri et al.’s [63] non-RCT, pre–post-ACT scores improved for cognitive restraint and quality-of-life rating for social life (medium effect sizes) and hunger, overall quality of life and quality-of-life ratings for body esteem and family relations (small effect sizes). However, body dissatisfaction showed a mean increase pre–post-ACT, with a small effect size. Disinhibition and physical comfort did not change pre–post-ACT.

##### Factors Associated with CYP-Reported Psychosocial Outcomes in CYP with Obesity

In Guerrini et al.’s [52] RCT, pre-ACT psychosocial outcomes (anxiety, depression, emotion dysregulation, experiential avoidance and fusion, and stress) were not significantly associated with emotional eating post-ACT.

##### Acceptability of Interventions in CYP with Obesity

Tronieri et al.’s [63] non-RCT assessed acceptability. Adolescents, including the one who dropped out, reported high acceptability on an adapted version of the Treatment Evaluation Inventory—Short Form [103]. They also gave high ratings for likelihood of enrolling in a similar programme in the future, if they wanted to lose more weight or maintain their weight, and reported frequent use of ACT skills.

##### Summary for CYP with Obesity

Findings from obesity studies provide mixed and limited evidence for the effectiveness of ACT on CYP-reported psychosocial outcomes. Across two studies, no significant effects were found for stress, while results for depression and experiential avoidance/fusion were inconsistent, with one RCT reporting no effects and a smaller non-RCT showing improvements in depression but worsening experiential avoidance. Other outcomes were assessed in single studies, with some evidence supporting improvements in food addiction, emotional eating, certain aspects of quality of life and cognitive restraint; however, results were often inconsistent or lacked between-group differences. In some cases, outcomes worsened, such as increases in anxiety and body dissatisfaction. Notably, all studies were rated as high/serious risk of bias. Methodological differences, including variation in measures, control groups (and lack of) and small sample sizes, may have contributed to these inconsistencies. Pre-intervention psychosocial factors were not associated with post-intervention emotional eating in one RCT. Acceptability assessed by one non-RCT supported ACT as well-received, with adolescents reporting high satisfaction and engagement with intervention skills.

**Table 6:** Summary of findings for obesity.

Study (Design)	Time Points	CYP-Reported Psycho Social Outcomes	Findings on Intervention Effects on CYP-Reported Psychosocial Outcomes	Factors Associated with ACT Intervention Effects on CYP-Reported Psychosocial Outcomes
Davoudi (2021) [51] (RCT)	Pre-intervention Post-intervention 12-week follow-up	Food addiction, YFAS	Food addiction showed significantly greater changes over time (pre–post-follow-up) in those receiving ACT than psychoeducation on individual advice (*p* < 0.001), with means being lower in ACT at both post-intervention and follow-up.	Not studied.
Guerrini Usubini et al. (2022) [52] (RCT)	Pre-intervention Post-intervention	Anxiety, subscale of DASS-21	For anxiety, a significant time–group interaction effect was reported (*p* = 0.031; *ƞ*^2^ = 0.02). Levels of anxiety significantly increased in both groups, with a higher increase in ACT+TAU than in TAU over time.	The groups (ACT+TAU vs. TAU only) did not significantly moderate the effect of pre-intervention anxiety (*p* = 0.543; *ƞ*^2^ = 0.01) or emotion dysregulation (*p* = 0.785; *ƞ*^2^ = 0.00) on emotional eating at post-intervention. Although the groups did significantly moderate the effect of pre-intervention depression (*p* = 0.018; *ƞ*^2^ = 0.15), experiential avoidance and fusion (*p* = 0.044; *ƞ*^2^ = 0.06) and stress (*p* = 0.043, *ƞ*^2^ = 0.10) on post-intervention emotional eating, in the ACT+TAU group pre-intervention depression, experiential avoidance and fusion and stress were not significantly associated with emotional eating at post-intervention.
		Depression, subscale of DASS-21	For depression, the time–group interaction effect (*p* = 0.784, *ƞ*^2^ = 0.000) was not significant.	
		Emotion dysregulation, DERS	For emotion dysregulation, the time–group interaction effect (*p* = 0.332, *ƞ*^2^ = 0.00) was not significant.	
		Emotional eating, subscale of DEBQ-EE	For emotional eating, the time–group interaction effect was not significant (*p* = 0.896, *ƞ*^2^ = 0.00), although a significant main effect of time (*p* = 0.010, *ƞ*^2^ = 0.01) was reported. This indicates emotional eating decreased pre–post-intervention in both ACT+TAU and TAU.	
		Experiential avoidance and fusion, AFQ-Y	For experiential avoidance and fusion, the time–group interaction effect (*p* = 0.299, *ƞ*^2^ = 0.00) was not significant.	
		Psychological wellbeing, PWB	For psychological wellbeing, the time–group interaction effect (*p* = 0.990, *ƞ*^2^ = 0.00) was not significant.	
		Stress, subscale of DASS-21	For stress, the time–group interaction effect (*p* = 0.844, *ƞ*^2^ = 0.00) was not significant	
Tronieri et al. (2019) [63] (RCT without a control group)	Pre-intervention Post-intervention	Body dissatisfaction, subscale of the EDI-3	Body dissatisfaction showed a mean increase pre–post-intervention (*d* = 0.31). *	Not studied.
		Cognitive restraint, subscale of EI	Cognitive restraint showed a mean increase pre–post-intervention (*d* = 0.76) *	
		Depression, PHQ-A	Depression showed a mean reduction pre–post-intervention (*d* = 1.20). *	
		Disinhibition, subscale of EI	Disinhibition shown no change pre–post-intervention (*d* = 0.06). *	
		Experiential avoidance, AFQ-Y	Experiential avoidance showed a mean increase pre–post-intervention (*d* = 0.43). *	
		Hunger, subscale of EI	Hunger showed a mean reduction pre–post-intervention (*d* = 0.42). *	
		Mindfulness, CAMM	Mindfulness showed no change pre–post-intervention (*d* = 0.04). *	
		Perceived stress, PSS-14	Perceived stress showed no change pre–post-intervention (*d* = 0.17). *	
		Quality of life (total), IWQOL-Kids	Overall quality-of-life rating showed a mean increase pre–post-intervention (*d* = 0.38). *	
		Quality-of-life body esteem, subscale of IWQOL-Kids	Quality-of-life rating for body esteem showed a mean improvement pre–post-intervention (*d* = 0.44). *	
		Quality-of-life family relations, subscale of IWQOL-Kids	Quality-of-life rating for family relations showed a mean improvement (*d* = 0.26). *	
		Quality-of-life physical comfort, subscale of IWQOL-Kids	Quality of life for physical comfort showed no change pre–post-intervention (*d* = 0.19). *	
		Quality-of-life social life, subscale of IWQOL-Kids	Quality-of-life rating for social life showed a mean increase pre–post-intervention (*d* = 0.82). *	
			Note: The study states that because this was a feasibility study; it was not powered to detect statistical significance. Therefore, tests of statistical significance were not conducted, and instead the focus was on effect sizes.	

Note. ACT = acceptance and commitment therapy. CYP = children and young people. RCT = randomised controlled trial. TAU = treatment as usual. Studies are ordered by design (RCT to non-RCT without control group). * = no *p*-value reported. Measures used: AFQ-Y = Avoidance and Fusion Questionnaire for Youths [104] Italian version [105]; CAMM = Child and Adolescent Mindfulness Measure [92]; DASS-21 = Depression Anxiety Stress Scales, Italian version [106]; DEBQ = Dutch Eating Behavior Questionnaire, Italian version [107]; DERS = Difficulties in Emotion Regulation Scale, Italian version [108]; EDI-3 = Eating Disorder Inventory 3 [109]; EI = Eating Inventory [110]; IWQOL-Kids = Impact of Weight on Quality of Life—Kids scale [111]; PHQ-A = Patient Health Questionnaire for Adolescents [112]; PSS-14 = Perceived Stress Scale [97]; PWB = Psychological Well-Being Scales, Italian version [113]; YFAS = Yale Food Addiction Scale [114].

#### 3.4.4. Cancer

##### Effectiveness on CYP-Reported Psychosocial Outcomes in CYP with Cancer

Outcome data from cancer studies are presented in Table 7. For anger, between-group analyses supported the effectiveness of ACT (to one-month follow-up) compared with a meeting offering a solution in one RCT by Asadi et al. [53]. For resilience and self-concept, the effects of ACT were also supported compared with NI in one non-RCT by Ebrahimi et al. [57]. Of note, effect sizes for all outcomes across both studies were large, and no dropouts were reported for both studies. However, both studies provided insufficient reporting of their methodology.

##### Factors Associated with Psychosocial Outcomes and Acceptability of Interventions in CYP with Cancer

Not studied.

##### Summary for CYP with Cancer

One RCT found ACT to be more effective than a solution-focused meeting in reducing anger up to one-month follow-up, while a non-RCT reported improvements in resilience and self-concept compared to no intervention. Both studies reported large effect sizes and no dropouts, indicating promising acceptability and impact. However, confidence in these findings is constrained by poor methodological reporting across studies. No research examined factors associated with psychosocial outcomes or the acceptability of interventions.

#### 3.4.5. Visual Impairment

##### Effectiveness on CYP-Reported Psychosocial Outcomes in CYP with Visual Impairment

Outcome data from the visual impairment study are presented in Table 8. For emotional maturity and its components, between-group analyses supported the effectiveness of ACT compared with empowering make-up sessions in one RCT by Mirmohammadi and Pourmohamadreza-Tajrishi [54], with large effect sizes. The effects of ACT were maintained at the eight-week follow-up. Although this RCT included an active control, details on the randomisation process were not reported.

##### Factors Associated with CYP-Reported Psychosocial Outcomes in CYP with Visual Impairment

Not studied.

##### Acceptability of Interventions in CYP with Visual Impairment

Mirmohammadi and Pourmohamadreza-Tajrishi [54] assessed acceptability using the Intervention Rating Profile Questionnaire (IPR-15) [118]. The social validity rating of ACT indicated it was acceptable to adolescents.

##### Summary for CYP with Visual Impairment

Evidence from one RCT suggests that ACT may be effective in improving psychosocial outcomes, specifically emotional maturity and its subscales, in CYP with visual impairment, with large and sustained effects observed at the eight-week follow-up. However, confidence in these findings is limited by high risk of bias, including insufficient reporting of the randomisation process. No studies examined factors associated with psychosocial outcomes. Acceptability data indicate that ACT is perceived as acceptable by adolescents, based on social validity ratings.

**Table 8:** Summary of findings for visual impairment.

Study (Design)	Time Points	CYP-Reported Psychosocial Outcomes	Findings on Intervention Effects on CYP-Reported Psychosocial Outcomes
Mirmohammadi and Pourmohamadreza-Tajrishi (2024) [54] RCT	Pre-intervention Post-intervention Eight-week follow-up	Emotional maturity (overall), EMS	Emotional maturity scores were significantly lower (indicating higher levels) in ACT than make-up empowering sessions post-intervention, after controlling for pre-test scores (*p* = 0.001, *ηp*^2^ = 0.90 ^†^), with the means indicating emotional maturity decreased only in ACT.
		Emotional progression, subscale of EMS	Emotional progression scores were significantly lower (indicating higher levels) in ACT than make-up empowering sessions post-intervention, after controlling for pre-test scores (*p* = 0.001, *ηp*^2^ = 0.59 ^†^), with the means indicating emotional maturity decreased only in ACT.
		Emotional stability, subscale of EMS	Emotional stability scores were significantly lower (indicating higher levels) in ACT than make-up empowering sessions post-intervention, after controlling for pre-test scores (*p* = 0.001, *ηp*^2^ = 0.69 ^†^), with the means indicating emotional maturity decreased only in ACT.
		Independence, subscale of EMS	Independence scores were significantly lower (indicating higher levels) in ACT than make-up empowering sessions post-intervention, after controlling for pre-test scores (*p* = 0.001, *ηp*^2^ = 0.68 ^†^), with the means indicating emotional maturity decreased only in ACT.
		Personality integration, subscale of EMS	Personality integration scores were significantly lower (indicating higher levels) in ACT than make-up empowering sessions post-intervention, after controlling for pre-test scores (*p* = 0.001, *ηp*^2^ = 0.92 ^†^), with the means indicating emotional maturity decreased only in ACT.
		Social adaptation, subscale of EMS	Social adaption scores were significantly lower (indicating higher levels) in ACT than make-up empowering sessions post-intervention, after controlling for pre-test scores (*p* = 0.001, *ηp*^2^ = 0.55 ^†^), with the means indicating emotional maturity decreased only in ACT.
			In the ACT group, there were no significant differences between post-intervention and follow-up for emotional maturity (*p* = 0.660, *d* = 0.22), emotional progression (*p* = 0.210, *d* = 0.02), emotional stability (*p* = 0.240, *d* = 0.08), independence (*p* = 0.749, *d* = 0.02), personality integration (*p* = 0.504, *d* = 0.02) and social adaptation (*p* = 0.489, *d* = 0.05).

Note. ACT = acceptance and commitment therapy. CYP = children and young people. RCT = randomised controlled trial. ^†^ = reported as ‘eta coefficients’. *ηp*^2^ inferred as effect size assessed due to the context of multivariate analysis and the reporting of variance explained in report. Measure used: EMS = Emotional Maturity Scale [119].

## 4. Discussion

This systemic review evaluated the effectiveness of ACT on CYP-reported psychosocial outcomes among CYP with LTCs. The factors associated with the effects and the quantitative acceptability of the included ACT interventions were also examined. Following a systematic literature search, 16 studies from 19 reports were included.

### 4.1. Effectiveness of ACT on CYP-Reported Psychosocial Outcomes

Many CYP-reported psychosocial outcomes were evaluated, encompassing mental health, emotional wellbeing, cognitive processes, interpersonal relationships, functioning and quality of life. All 15 studies that assessed statistical significance reported significant improvements in at least one CYP-reported psychosocial outcome [46,48,49,50,51,52,53,54,55,57,58,59,60,61,62]. Notably, eight of these studies reported significant improvements across all CYP-reported psychosocial outcomes assessed, with effect sizes ranging from small to large [49,50,51,53,54,57,60,62]. Collectively, these findings provide preliminary support for the potential transdiagnostic utility of ACT in CYP with LTCs, although conclusions remain tentative given the methodological limitations of the evidence base.

It is noteworthy that significant effects on all CYP-reported psychosocial outcomes were found only in the cancer and visual impairment studies; however, these LTC groups included fewer studies and assessed a narrower range of outcomes. Although most CYP-reported psychosocial outcomes demonstrated significant effects of ACT among CYP with chronic pain, diabetes and obesity, a minority did not. In most cases, these non-significant results were limited to one or two outcomes per study.

Mixed findings within LTC groups were reported for a small number of CYP-reported psychosocial outcomes. Depression, the most frequently assessed outcome, was examined in ten studies across chronic pain, diabetes and obesity [46,47,48,50,52,58,59,60,61,63], but findings within each LTC were inconsistent. Similarly, insomnia in chronic pain [58,61] and experiential avoidance in obesity [52,63] showed mixed results. While notable methodological heterogeneity between studies may have contributed to such inconsistencies, all were limited by high/serious risk of bias ratings. Only two outcomes worsened pre–post-ACT, each in one obesity study: anxiety [52] and body dissatisfaction [63]; however, the latter was not tested for statistical significance.

A variety of ACT intervention delivery structures, doses, formats, methods and contents showed significant effects. This suggests adaptability in how ACT can be implemented within paediatric LTC settings, including potentially less resource-intensive formats.

### 4.2. Factors Associated with Psychosocial Outcomes

Factors associated with the effects of the evaluated ACT interventions were considered in seven studies (five chronic pain, one diabetes and one obesity) [48,52,55,58,59,60,64]. Although psychological flexibility is theorised to underpin ACT, evidence supporting its role as a mechanism of change in CYP with LTCs remains limited and mixed. One study found no mediating effect of adolescent diabetes-related psychological flexibility on anxiety [48], differing from findings in other clinical samples, including adult LTC populations [120,121,122]. However, in another study, changes in parent psychological flexibility were positively associated with changes in adolescent pain acceptance [60]. One further study assessing individual psychological flexibility processes found that higher acceptance in CYP statements early in ACT treatment was associated with greater decreases in pain interference, whereas cognitive defusion, values and committed action were not [64]. Taken together, these findings provide only preliminary and inconsistent support for psychological flexibility as a mechanism of change in this population.

Significant mediators, namely pain reactivity on pain interference and depression and pain-impairment beliefs on depression [55], and moderators, namely pain risk on improvements in pain catastrophising, depression and pain frequency [60], were also found. Other factors associated with the effects of ACT were autistic traits and ADHD [58]. However, these factors were only assessed in one study each, with several other factors also showing non-significant associations.

### 4.3. Acceptability of ACT Interventions

Quantitative data on the acceptability of the included interventions were provided for three studies (in diabetes, obesity and visual impairment) [48,54,56,63]. Scales (including two standardised measures) were utilised, with high acceptability/satisfaction reported by CYP across studies.

One study considered satisfaction with intervention length, with most participants being satisfied [48,56]. Of other acceptability questions asked, all but one adolescent reported they would recommend the intervention to others [48,56], ratings were high for the likelihood of signing up to a similar programme in the future if warranted [63] and all participants reported perceived benefits [56].

### 4.4. Limitations

The findings of this systematic review should be viewed in the context of several limitations. Overall, the included studies had a small, combined sample size and were predominantly female, with limited or no reporting of participant ethnicity. Studies were also conducted in a limited number of countries and included only five LTCs. These sample characteristics substantially limit the generalisability of findings to the wider population of CYP with LTCs across diverse cultural and healthcare contexts, highlighting the need for future research involving more diverse and representative samples.

Although the broad search strategy was intended to maximise sensitivity across diverse LTC populations, the exclusion of LTC-specific terms may have affected study identification and risked missing relevant studies where physical health conditions were not clearly described or indexed. In addition, most screening was conducted by a single reviewer, with only a subset independently checked, introducing potential selection bias and possible inconsistencies despite reported inter-rater agreement. Future reviews should consider more comprehensive search strategies alongside full dual independent screening to strengthen methodological rigour.

Most RCTs were small-scale or pilots, and only one non-RCT had a control group. Few studies used active controls, making it difficult to ascertain whether effects were due to ACT or non-intervention specific factors (e.g., therapeutic alliance) [123]. Most studies also had no or short follow-ups, limiting assessment regarding sustained effects. The synthesis was limited by the high heterogeneity of included studies, rendering quantitative pooling (i.e., meta-analysis) inappropriate. Such heterogeneity also limited the ability to meaningfully compare studies or identify which intervention components were most strongly associated with effects in psychosocial outcomes. Moreover, most CYP-reported psychosocial outcomes were examined in only one study, and effect sizes for several outcomes were not reported and could not be calculated from the available data. Thus, conclusions surrounding the effects of ACT remain tentative. Conclusions on factors associated with these effects and acceptability are also limited given the small number of studies reporting such data. In addition, the narrative synthesis was necessarily descriptive, with limited capacity to formally weight evidence, compare effect sizes, or systematically examine patterns across studies of differing methodological quality. While some variation in intervention characteristics was noted, more detailed exploration of heterogeneity (e.g., treatment dose, which varied substantially across studies including one intensive 90 h intervention, parental involvement, group versus individual delivery and in-person versus telehealth formats) was not feasible given the small number of studies and variability in reporting.

Almost all studies had high/serious risk of bias, along with poor reporting of key methodological details, which may inflate effect sizes and undermine the reliability and validity of the findings. However, these ratings partly reflect the quality assessment tools used. Although Cochrane recommends RoB 2 and ROBINS-I for systematic reviews evaluating interventions [43,44], some criteria (e.g., blinding) are difficult to meet in psychological interventions requiring active participation [124]. Some criteria also misaligned with the current systematic review; for example, outcome measurement domains received poorer ratings due to the inclusion criteria of the systematic review (i.e., CYP-reported psychosocial outcomes). While preliminary findings suggest ACT may hold promise for CYP with LTCs, the consistently high risk of bias and methodological limitations across studies mean conclusions regarding effectiveness remain tentative and require evaluation in higher-quality research.

### 4.5. Clinical Implications and Future Directions

This systematic review provides preliminary, tentative support for the effectiveness and acceptability of ACT in CYP with LTCs, delivered face-to-face and online and in different formats (e.g., individual and group-based). While these findings suggest ACT may have transdiagnostic potential and flexibility for adaptation within paediatric LTC contexts, they should be interpreted cautiously and do not yet justify routine clinical implementation. At present, ACT may be most appropriately considered as a potentially adaptable psychosocial approach that could be incorporated within broader multidisciplinary or stepped-care paediatric services where appropriate, rather than as a standardised intervention or first-line treatment. This aligns conceptually with stepped-care models in paediatric psychology, such as the Pediatric Psychosocial Preventative Health Model (PPPHM) [125], which emphasises matching intervention intensity to patient and family psychosocial need. Given the variability in intervention intensity, format, content and delivery across studies, alongside limited evidence regarding which components are most effective, practical implementation would likely require careful consideration of developmental appropriateness, LTC-specific needs, clinician training, service capacity and accessibility across delivery methods. Encouragingly, research on ACT studies in CYP with LTCs are increasing, with half of the included studies published in the past five years. Important uncertainties remain for practice, including variability in intervention intensity, format and content, and limited evidence regarding which components are most effective. In addition, the high risk of bias and limited long-term follow-up across studies constrain confidence in the robustness and sustainability of effects. There is also insufficient high-quality evidence to support recommendations for the use of ACT within the specific LTC groups represented in this review at this stage. Although findings suggest ACT can be delivered in varied ways, further research is needed before conclusions can be drawn regarding optimal treatment dose, parental involvement, mode of delivery or suitability for particular clinical subgroups.

To broaden the evidence base, it is important for future research to evaluate ACT among CYP with other LTCs, seeking more diverse samples. Consistency in studies should be sought to enable meta-analyses to be performed. To better understand ACT treatment processes, assessments of psychological flexibility and other potential mediators and moderators should also be included in future trials. Acceptability should also be considered to help support intervention engagement and satisfaction. This could include qualitative findings to offer a richer understanding of acceptability. Theoretical frameworks, such as the Theoretical Framework of Acceptability for healthcare interventions [126], could be used to guide the assessment and interpretation of acceptability. To widen applicability, future systematic reviews should consider such findings on acceptability and the inclusion of a broader range of outcomes, such as LTC-related outcomes (e.g., physical symptoms), parent/carer-reported CYP outcomes and the wider impact on families (e.g., parents/carers and siblings). Future systematic reviews with a larger and more homogeneous evidence base should also consider more structured comparative or meta-analytic approaches to better evaluate how intervention characteristics (e.g., dose, delivery format and parental involvement) may be associated with outcomes.

Taken together, the findings on ACT among CYP with LTCs remain limited. To build upon the current evidence base, large-scale, methodologically rigorous and well-reported RCTs with active control conditions and long-term follow-ups are warranted. Such research could inform the development of care pathways and clinical guidelines for CYP with LTCs.

## 5. Conclusions

This systematic review of 16 eligible studies across 19 reports identified methodologically limited but promising evidence on the effectiveness of ACT interventions for CYP-reported psychosocial outcomes among CYP with LTCs. Although significant improvements for a range of outcomes were reported across studies, findings were inconsistent, and almost all studies were rated as having high/serious risk of bias, meaning conclusions regarding effectiveness remain tentative. Seven studies explored factors associated with intervention effects, with mixed and inconclusive results, while acceptability, although generally supported, was assessed in only three studies. Overall, there is a clear need for more methodologically rigorous research, including well-powered RCTs with long-term follow-ups in diverse populations and routine assessment of acceptability, to better establish the role of ACT in psychosocial care pathways for CYP with LTCs.

## Figures and Tables

**Table 1:** Inclusion criteria using Patient, Intervention, Comparison and Outcome (PICO) framework [38].

Component	Inclusion Criteria
General	Quantitative experimental design (randomised and non-randomised designs, given the limited evidence base), including mixed-method studies with a quantitative component. Published in a peer-reviewed journal (i.e., not a dissertation or non-peer reviewed conference abstract or pre-print). Published in English. Any publication year.
Population	Participants were all ≤18-years-old (or sub-analyses for ≤18-year-olds). Participants were all diagnosed with LTC(s). LTCs were defined as physical health conditions that typically persist for at least three months, require medical care and are associated with functional limitations [39].
Intervention	Any ACT-based intervention delivered to CYP. This may be with or without parent(s)/carer(s) involvement. ACT-based intervention only (or sub-analyses for ACT-based component if used in conjunction with other psychological/psychosocial interventions).
Comparator	Reports’ comparisons in outcomes at pre-intervention and post-intervention. RCTs should additionally report comparisons between an intervention and control/comparator group.
Outcome	Reports’ CYP-reported measure of any psychosocial outcome (e.g., anxiety, depression, psychological distress and coping).

Note. CYP = children and young people. LTC = long-term physical health condition. RCT = randomised controlled trial.

**Table 2:** Study designs and participant characteristics of included studies.

Study	Design	Control/ Comparator	Country	LTC	Participants					
					ACT			Control/Comparator		
					Number	Age in Years	Gender	Number	Age in Years	Gender
**Chronic Pain**										
Kanstrup et al. (2016) [46]	RCT (pilot).	Compared two delivery formats of ACT intervention (group and individual), with neither framed as the control/comparator.	Sweden.	Chronic pain (for more than six months).	*N* = 24 (randomised). ^			*N* = 24 (randomised). ^		
					*n* = 12 (post-intervention).	*M* (*SD*): 16.3 (1.5). Range: 14–18 (overall).	11 (91.7%) females (91.7%), one (8.3%) male.	*n* = 18 (post-intervention).	*M* (*SD*): 15.8 (1.6). Range: 14–18 (overall).	13 (72.2%) females, five (27.7%) males.
Wicksell et al. (2009) [47]	RCT.	TAU (multidisciplinary treatment and amitriptyline. Approach guided by biobehavioural model of longstanding pain, with input from physician, physiotherapist, psychiatrist and psychologist).	Sweden.	Chronic pain (idiopathic, more than three months).	*N* = 16 (randomised).	*M* (*SD*): 14.8 (2.4) (overall) Range: 10.8–18.1 (overall).	25 (78.1%) females, seven (21.9%) males (overall).	*N* = 16 (randomised).	*M* (*SD*): 14.8 (2.4) (overall) Range: 10.8–18.1 (overall).	25 (78.1%) females, seven (21.9%) males (overall).
					*n* = 15 (post-intervention), *n* = 14 (one-month follow-up) and *n* = 13 (two-month follow-up). ^			*n* = 14 (post-intervention), *n* = 11 (one- and two-month follow-ups). ^		
Secondary analysis reported in Wicksell et al. (2011) [55]					*n* = 15 (post-intervention).	*M* (*SD*): 14.8 (2.4). Range: 10.8–18.1.	23 (76.7%) females, seven (23.3%) males.	Not included in secondary analysis.		
Balter et al. (2021) [58]	Non-RCT without control group.	None.	Sweden.	Chronic pain (for more than six months).	*N* = 47 (recruited, no dropout, although some missing data).	*M* (*SD*):14.8 (2.2). Range: 9.5–17.9.	33 (70%) females.	None.		
Kemani et al. (2018) [59]	Non-RCT without control group.	None.	England/UK.	Chronic pain.	*N* = 187 (recruited). ^			None.		
					*n* = 164 (pre-intervention).	*M* (*SD*): 15.5 (1.8). Range: 11.3–18.9.	127 (77.4%) females.			
					*n* = 164 (post-intervention). ^ *n* = 107 (three-month follow-up). ^					
Vuorimaa et al. (2019) [60]	Non-RCT without control group (feasibility study).	None.	Finland.	Chronic pain (idiopathic recurrent musculoskeletal pain, persistent pain over three months).	*N* = 32 (recruited).	*M* (*SD*): 14.4 (1.3). Range: 13–17.	26 (81%) females.	None.		
					*n* = 24 (six- and 12-month follow-ups from pre-intervention). ^					
Zetterqvist et al. (2020) [61]	Non-RCT without control group (pilot).	None.	Sweden.	Chronic pain (duration ≥ three months).	*N* = 28 (recruited).	*M* (*SD*) = 15.43 (1.26). Range = 13–17.	100% females.	None.		
					*n* = 23 (post-intervention). ^					
					*n* = 20 (four-month follow-up, completed at 17–25 weeks). ^					
Secondary analysis reported in Gentili et al. (2024) [64]					No separate demographic information for participants in secondary analyses.			None.		
**Diabetes**										
Alho et al. (2022) [48]	RCT (pilot).	TAU (included paediatric clinic visits once every three months and meeting with healthcare professionals, including a nurse and a doctor). Offered the same intervention after the post-measurements (final time point).	Finland.	Diabetes (type 1, with HbA1c levels above 7.5%).	*N* = 36 (randomised).	*M* (*SD*): 13.44 (13.44). Range: 12–16 (overall).	24 (67%) females, 12 (33%) males.	*N* = 36 (randomised).	*M* (*SD*): 13.36 (1.22). Range: 12–16 (overall).	21 (58%) females, 15 (42%) males.
					*n* = 31 (pre-intervention).	*M* (SD): 13.39 (1.12). Range: 12–16 (overall).	22 (71%) females, nine (29%).	*n* = 29 (pre-intervention).	*M* (*SD*): 13.48 (1.30). Range: 12–16 (overall).	16 (55%) females, 13 (45% males).
					*n* = 28 (post-intervention). ^			*n* = 27 (post-intervention). ^		
Secondary analysis reported in Alho et al. (2024) [56]					*n* = 28 (post-intervention).	Range: 12–16 (overall). States 15 participants were 12–13 and 13 participants were 14–16.	Not reported.	None.		
Ataie Moghanloo et al. (2015) [50]	RCT.	NI.	Iran.	Diabetes (type 1 or type 2, for at least one year).	*N* = 20 (randomised). ^			*N* = 20 (randomised). ^		
					*n* = 17 (post-intervention).	*M* (*SD*): 10.35 (2.91). Range: 7–15 (overall).	Eight (47.1%) males, nine (52.9%) females.	*n* = 17 (post-intervention).	*M* (*SD*): 10.59 (3.16). Range: 7–15 (overall).	Nine (52.9%) males, eight (47.1%) females.
Moazzezi et al. (2015) [49]	RCT.	NI.	Iran.	Diabetes (type 1 or type 2, for at least one year).	*N* = 20 (randomised). ^			*N* = 20 (randomised). ^		
					*n* = 18 (post-intervention).	*M* (*SD*): 11.44 (2.59). Range: 7–15 (overall).	12 (66.7%) males, four females (22.2%), two (11.1%) not reported.	*n* = 18 (post-intervention).	*M* (*SD*): 9.72 (2.37). Range: 7–15 (overall).	Nine (50%) males, seven (38.9%) females, two (11.1%) not reported.
Stefanescu et al. (2024) [62]	Non-RCT without control group.	None.	Romania.	Diabetes (type 1, for at least one year).	*N* = 57 (recruited). ^			None.		
					*n* = 55 (post-intervention).	*M* (*SD*): 14.14 (2.26) Range: 10–18.	67% females.			
**Obesity**										
Davoudi (2021) [51]	RCT.	Psychoeducation individual advice (12 weekly 90 min sessions, delivered by person with clinical psychology masters and training in individual and group counselling for adolescent disorders).	Iran.	Obesity (BMI ≥ 95th percentile for age and gender).	*N* = 25 (randomised).	*M* (*SD*): 15.22 (1.74). Range: 12–18 (overall).	100% females.	*N* = 25 (randomised).	*M* (*SD*): 15.1 (2.22). Range: 12–18 (overall).	Gender: 100% females.
					*n* = 22 (post-intervention and 12-week follow-up). ^			*n* = 24 (post-intervention) and *n* = 23 (12-week follow-up). ^		
Guerrini Usubini et al. (2022) [52]	RCT.	TAU (received standard three-week in-hospital multidisciplinary rehabilitation program for weight loss and the standard psychological assessment and support for hospitalisation. This included dietary input, nutritional counselling, physical activity program with training and psychological counselling provided by clinical psychologist).	Italy.	Obesity (BMI > 97th percentile for age and gender).	*N* = 25 (randomised). ^			*N* = 24 (randomised). ^		
					*n* = 17 (post-intervention).	*M* (*SD*): 15.5 (1.37). Range: 13–17 (overall).	13 (76.5%) females, four (23.5%) males.	*n* = 17 (post-intervention).	*M* (*SD*): 15.6 (1.06). Range: 13–17 (overall).	14 (82.4%) females, three (17.6%) males.
Tronieri et al. (2019) [63]	Non-RCT without control group (feasibility study).	None.	USA.	Obesity (BMI ≥ 95th percentile for age and gender).	*N* = 7 (recruited).	*M* (*SD*): 13.7 (1.7). Range: 12–16.	Six (85.7%) females.	None.		
					*n* = 6 (post-intervention).	*M* (*SD*): 13.7 (1.9). range: 13.7 (1.9), 12–16.	Five (83.3%) females.			
**Cancer**										
Asadi et al. (2016) [53]	RCT.	A meeting without offering a solution.	Iran.	Cancer.	*N* = 15 (randomised, no dropout).	*M* (*SD*): not reported. Range: 9–14 (overall).	Not reported.	*N* = 15 (randomised, no dropout).	*M* (*SD*): not reported. Range: 9–14 (overall).	Not reported.
Ebrahimi et al. (2022) [57]	Non-RCT with control.	NI.	Iran.	Leukaemia (with at least a one-year history of the illness).	*N* = 15 (randomised, no dropout).	*M* (*SD*): not reported. Range, 8–14 (overall).	Not reported.	*N* = 15 (randomised, no dropout).	*M* (*SD*): not reported. Range, 8–14 (overall).	Not reported.
**Visual impairment**										
Mirmohammadi and Pourmohamadreza-Tajrishi (2024) [54]	RCT.	Make-up empowering sessions in the complex. The content of the ACT intervention was briefly held for the control group in three sessions after the end of the research process and follow-up period.	Iran.	Visual impairment (from low vision to total blindness).	*N* = 14 (randomised).	*M* (*SD*): 16.17 (not reported). Range: 14–18 (overall).	100% females.	*N* = 14 (randomised).	*M* (*SD*): 16.22 (not stated). Range: 14–18 (overall).	100% females.
					*n* = 12 (post-intervention and eight-week follow-up). ^			*n* = 14 (post-intervention). ^		

Note. ACT = acceptance and commitment therapy. LTC = long-term physical health condition. NI = no intervention. RCT = randomised controlled trial. TAU = treatment as usual. Studies ordered by (i) LTC (most to least number of studies) and (ii) design (RCTs, non-RCTs with control group and non-RCTs without control group). Participant numbers reported only at time points for which studies provided this information. ^ = no separate demographic data reported.

**Table 3:** Acceptance and commitment therapy intervention characteristics of included studies.

	ACT Intervention Contents (Core Processes of Psychological Flexibility in CYP Sessions Reported)	Parental Involvement	Setting	Profession of Facilitator	Delivery		
					Structure and Dose	Format	Method
**Chronic pain**							
Kanstrup et al. (2016) [46]	Acceptance, cognitive defusion, committed action and values. ^ Note: Same intervention as Balter et al. (2021) [58].	Combination of joint and parallel parent sessions. Parent program aimed to enhance parents’ ability in supporting their child to improve their functioning. Included pain education, values clarification and acceptance skills to manage personal distress in relation to their child’s pain.	Tertiary pain specialist clinic.	Team of psychologists, pain physicians and physiotherapist. Most sessions conducted by a psychologist.	14 sessions for CYP. Session 12 was a joint session for CYP and parents. Three parent sessions held in parallel to CYP (for sessions three, six and 11). Group sessions were two hours and individual sessions were 45 min.	12 group (four groups, two-four participants per group) and 18 individual sessions. Short summary individually provided for each missed session.	Face-to-face.
Wicksell et al. (2009, 2011) [47,55]	Acceptance, cognitive defusion, committed action, self as context and values. Present moment inferred.	Separate parent sessions. Parent work emphasised the shift from symptom alleviation to valued life and operant mechanisms and exposure principles. Parental difficulties were addressed using the same psychological flexibility processes as in CYP sessions.	Not stated. Recruited from hospital.	Psychologists and physician.	Approximately 10 sessions (one hour, once a week) for CYP. One or two parent sessions (90 min).	Individual.	Face-to-face.
Balter et al. (2021) [58]	Acceptance, cognitive defusion, committed action and values. ^ Note: Same intervention as Kanstrup et al. (2016) [46].	Combination of joint and parallel parent sessions. Parent program aimed to enhance parents’ ability in supporting their child to improve their functioning. Included pain education, values clarification and acceptance skills to manage personal distress in relation to their child’s pain.	Not stated. Recruited via a tertiary pain clinic.	Psychologists.	14 sessions for CYP and one joint session for CYP and parents. Three parent sessions held in parallel to CYP. All sessions were two hours each.	Group.	Face-to-face.
Kemani et al. (2018) [59]	Acceptance, cognitive defusion, committed action, present moment, self as context and values.	Parents participated in most CYP sessions and modelled intervention skills. Parent-only group input focused on applying the skills learned in the previous session to manage challenging parenting situations. Parents also had some keyworker sessions, either with or without the adolescent.	Tertiary national specialist centre.	Interdisciplinary (medics, psychologists, physiotherapists, occupational therapists and nurses). ACT framework consistently applied throughout.	Approximately 90 h during a three-week period for CYP: 34 h of physical conditioning, 22 h of psychology, 15 h of activity management and 15 hours of mixed sessions. Parents participated in sessions, except for a four-day period mid-intervention when adolescents worked independently, and parents had three hours of a parent-only group. Structure/dose of parent keyworker sessions, with or without adolescent, not specified.	Mix of individual and group. CYP received, on average, three hours of individual input (i.e., majority were group-based, six per group).	Face-to-face.
Vuorimaa et al. (2019) [60]	Acceptance, committed action, present moment and values. Cognitive defusion and self as context inferred.	Parallel parent sessions. Parent sessions focused on developing pain-related problem-solving skills in the context of their child’s pain and managing health-related anxiety. Parents supported CYP with active and flexible strategies for coping with pain.	Paediatric rheumatology outpatient clinic.	Multidisciplinary team (psychologist, physiotherapist, paediatric rheumatologist and nurse).	10 days of sessions at the outpatient clinic for CYP. Intervention modules one and two occurred one and two months after the pre-intervention assessment. Parent group sessions were held in parallel.	Group (five-six per group).	Face-to-face.
Zetterqvist et al. (2020) [61], secondary analysis (Gentili et al., 2024) [64]	Acceptance, cognitive defusion, committed action, present moment and values. ^	Parents had access to seven parent modules (parent program), including acceptance, cognitive defusion, commitment action and values. Exercises related to content in CYP modules. Parents also received feedback from therapist via platform and in the second intake (of two) parents had a scheduled phone call (contents of feedback and phone calls not specified).	Internet-delivered. Conducted at a tertiary pain clinic in an urban setting and self-recruited from community via newspaper adverts and social media.	Psychologists.	Eight weeks of access to four CYP modules. CYP received feedback a minimum of every other weekday from therapist, via platform. In the second intake (of two), phone call scheduled at week two/three. Parents had eight weeks of access to seven modules; received feedback at least once a week; and in the second intake (of two), a phone call was scheduled at week two/three.	Individual.	Internet-delivered.
**Diabetes**							
Alho et al. (2022) [48], secondary analysis (Alho et al., 2024) [56]	Acceptance, cognitive defusion, committed action, present moment, self as context and values.	Parents joined start of first and last session to receive information regarding the intervention procedures.	Hospital.	Psychologist.	Five sessions (1.5 h, once a fortnight).	Group (five-seven per group).	Face-to-face.
Ataie Moghanloo et al. (2015) [50]	Acceptance, cognitive defusion, committed action, self as context and values. ^ Note: Same intervention as Moazzezi et al. (2015) [49].	None reported.	Not stated. Recruited from the Diabetes Association.	Psychologist.	10 sessions (90 min, once a week).	Group.	Face-to-face.
Moazzezi et al. (2015) [49]	Acceptance, cognitive defusion, committed action, self as context and values. ^ Note: Same intervention as Ataie Moghanloo et al. (2015) [50].	None reported.	Not stated. Recruited from the Diabetes Association.	Not reported.	10 sessions (90 min, once a week).	Group.	Face-to-face.
Stefanescu et al. (2024) [62]	Cognitive defusion, committed action, present moment and values. Committed action interred. ^	None reported.	Hospital (for face-to-face sessions).	Clinical psychologist.	Four sessions (50 min each, once per week).	Individual.	33% face-to-face, 67% online via Zoom.
**Obesity**							
Davoudi (2021) [51]	Acceptance, cognitive defusion, committed action, present moment, self as context and values.	Parents participated in first two sessions. Included work on creative helplessness and parental control strategies.	Not stated. Recruited via nutrition clinics and medical offices.	Clinical psychology masters.	12 sessions (90 min, once a week). First two sessions were joint sessions for CYP and parents.	Group.	Face-to-face.
Guerrini Usubini et al. (2022) [52]	Acceptance, cognitive defusion, committed action, present moment, self as context and values.	None reported.	Hospital (specialist clinical centre).	Clinical psychologist.	Three sessions (one hour, once a week).	Individual.	Face-to-face.
Tronieri et al. (2019) [63]	Acceptance, cognitive defusion, present moment and values. Committed action inferred. ^	Parallel parent sessions. Included supporting their child with healthy changes (e.g., modelling healthy behaviour), praising healthy choices (but not criticising unhealthy ones) and learning strategies through exercises (e.g., identifying and behaving in line with parenting values, accepting emotional experiences, defusing from thoughts and willingness).	Urban university medical centre.	Psychologist and registered dietician led simultaneous group sessions.	16 sessions (60–90 min, once a week). CYP and their parents/guardians attended separate groups.	Group. Make-up visit individually provided for each missed session.	Face-to-face.
**Cancer**							
Asadi et al. (2016) [53]	Medical consulting based on ACT. No details on content.	None reported.	Not stated. Recruited from hospital.	Counsellor.	Three sessions (two hours each). Between the final session and the follow-up, a weekly phone call with counsellor was provided to maintain contact and taper the intervention.	Group (five per group).	Face-to-face.
Ebrahimi et al. (2022) [57]	Contents/core processes not reported in English (translation deemed unsuitable, as psychological flexibility processes may not translate accurately due to lack of direct linguistic equivalents and reliance on culturally embedded metaphors).	None reported.	Hospital.	Not reported.	Six sessions (one-hour each).	Group.	Face-to-face.
**Visual impairment**							
Mirmohammadi and Pourmohamadreza-Tajrishi (2024) [54]	Acceptance, cognitive defusion, committed action, present moment, self as context and values.	None reported.	Specialist educational complex.	Not reported.	10 sessions (one hour, once a week).	Group (seven per group).	Face-to-face.

Note. ACT = acceptance and commitment therapy. CYP = children and young people. ^ = the report did not explicitly mention the remaining psychological flexibility process(es).

**Table 4:** Summary of findings for chronic pain.

Study (Design)	Time Points	CYP-Reported Psychosocial Outcomes	Findings on Intervention Effects on CYP-Reported Psychosocial Outcomes	Factors Associated with ACT Intervention Effects on CYP-Reported Psychosocial Outcomes
Kanstrup et al. (2016) [46] (RCT)	Pre-intervention Mid-intervention Post-intervention	Depression, CES-DC	Depression significantly decreased pre–post-intervention for both groups combined (*p* = 0.004; *r* = 0.37), with 39% of adolescents showing a clinically significant reduction. Pre–mid-intervention changes were not significant (*p* = 0.213), but mid–post-intervention changes were (*p* < 0.001).	Not studied.
		Pain intensity (rated 0–6)	Pain intensity did not significantly change pre–post-intervention for both groups combined (*p =* 0.346, *r* = -.13). Fifteen percent of adolescents showed a clinically significant reduction. Both pre–mid-intervention (*p* = 0.697) and mid–post-intervention changes (*p* = 0.255) were not significant.	
		Pain interference, PII	Pain interference significantly decreased pre–post-intervention for both groups combined (*p* < 0.001; *r* = 0.51), with 47% of adolescents showing a clinically significant reduction. Pre–mid-intervention changes were not significant (*p* = 0.026), but mid–post-intervention changes were (*p* = 0.002).	
		Pain reactivity, PRS	Pain reactivity significantly decreased pre–post-intervention for both groups combined (*p* < 0.001; *r* = 0.49), with 48% of adolescents showing a clinically significant reduction. Pre–mid-intervention changes were not significant (*p* = 0.362), but mid–post-intervention changes were (*p* < 0.001).	
		Psychological inflexibility, PIPS	Psychological inflexibility significantly decreased pre–post-intervention for both groups combined (*p* < 0.001; *r* = 0.59), with 63% of adolescents showing a clinically significant reduction. Pre–mid-intervention changes were not significant (*p* = 0.027) but mid–post-intervention changes were (*p* < 0.001).	
			Between-group analyses: There were no significant differences between the conditions (individual ACT and group ACT) in any of the outcome variables at any of the time points (*p* = 0.109 to 1.00). There were no significant differences between group and individual ACT in the number of adolescents with clinically significant pre-to-post-intervention improvements for any of the variables (*p* = 0.083 to 1.00). A trend toward a significant difference was indicated for depression (*p* = 0.028), with more adolescents in the individual condition reporting clinically significant reductions in depression. Note: A conservative significance level of *p* < 0.01 had been set to take into account multiple comparisons.	
Wicksell et al. (2009, 2011) [47,55] (RCT)	Pre-intervention Post-intervention One-month follow-up Two-month follow-up	Depression, CES-DC	Depression did not significantly change over time in ACT (*p* = 0.063; *ηp*^2^ = 0.18) or TAU (*p* = 0.568; *ηp*^2^ = 0.03). Between-group differences were not significant post-intervention (*p* = 0.055; *ηp*^2^ = 0.12) or when including follow-ups (*p* = 0.088; *ηp*^2^ = 0.10).	Post-ACT pain-impairment beliefs mediated changes in pre-ACT measures of depression to both follow-up one (95% CI = 1.75 to 14.59) and two (95% CI = 2.46 to 26.55). Post-ACT pain-impairment beliefs mediated changes in pre-ACT pain interference to follow-up one (90% CI = 0.00 to 2.40) but not two (95% CI = −0.41 to 3.18). Post-ACT pain reactivity mediated changes in pre-ACT measures of pain interference and depression to both follow-up one (pain interference, 95% CI = 0.08 to 3.01; depression, 95% CI = 0.01 to 14.77) and two (pain interference, 95% CI = 0.17 to 4.32; depression, 90% CI = 3.52 to 31.96). No significant mediation effects were found for post-ACT measures of internalising/catastrophising, kinesiophobia, pain intensity or self-efficacy measured using SES. Significant mediating effects were analysed further. Post-ACT pain-impairment beliefs were not significantly related to depression change scores from pre to follow-up one when controlling for depression post-ACT (*p* = 0.107). However, pain-impairment beliefs did significantly predict change to follow-up two (*p* = 0.028), with pain-impairment beliefs at post-ACT explaining 33% of the variance when controlling for post-ACT depression. Post-ACT pain reactivity significantly predicted changes in pain interference at both follow-up one (56% variance, *p* = 0.002) and follow-up one (54% variance, *p* = 0.003), after controlling for post-ACT pain interference. For depression, pain reactivity explained 21.1% of the variance in change to follow-up one (*p* = 0.090), but not at follow-up two (*p* = 0.111).
		Functional disability, FDI	Functional disability significantly decreased over time in ACT (*p* = 0.002; *ηp*^2^ = 0.38) and TAU (*p* = 0.049; *ηp*^2^ = 0.21). Between-group differences were not significant post-intervention (*p* = 0.944; *ηp*^2^ = 0.00) or when including follow-ups (*p* = 0.474; *ηp*^2^ = 0.02).	
		Internalising/catastrophising, subscale of PCQ	Internalising/catastrophising did not significantly change over time in ACT (*p* = 0.051; *ηp*^2^ = 0.19) or TAU (*p* = 0.106; *ηp*^2^ = 0.14). Between-group differences were not significant post-intervention (*p* = 0.622; *ηp*^2^ = 0.01) or when including follow-ups (*p* = 0.916; *ηp*^2^ = 0.00).	
		Kinesiophobia, TSK	Kinesiophobia significantly decreased over time in ACT (*p* < 0.001; *ηp*^2^ = 0.56) and TAU (*p* < 0.001; *ηp*^2^ = 0.42). Between-group differences were significant post-intervention, with significantly greater reductions in ACT (*p* = 0.010; *ηp*^2^ = 0.21), but not when including follow-ups (*p* = 0.052; *ηp*^2^ = 0.12).	
		Mental quality of life, subscale of SF-36	Mental quality of life significantly increased over time in ACT (*p* = 0.027; *ηp*^2^ = 0.22), but not TAU (*p* = 0.993; *ηp*^2^ = 0.00). Between-group differences were significant post-intervention, with significantly greater increases in ACT (*p* = 0.033; *ηp*^2^ = 0.15), but not when including follow-ups (*p* = 0.067; *ηp*^2^ = 0.11).	
		Pain-impairment beliefs, PAIRS	Pain-impairment beliefs significantly decreased over time in ACT (*p* = < 0.001; *ηp*^2^ = 0.47) and TAU (*p* = < 0.001; *ηp*^2^ = 0.33). Between-group differences were significant post-intervention, with significantly greater reductions in ACT (*p* = 0.002; *ηp*^2^ = 0.29), and when follow-ups were included (*p* = 0.007; *ηp*^2^ = 0.23).	
		Pain intensity (rated 0–10)	Pain intensity significantly decreased over time in ACT (*p* = 0.004; *ηp*^2^ = 0.35) but not TAU (*p* = 0.251; *ηp*^2^ = 0.09). Between-group differences were significant post-intervention, with significantly greater reductions in ACT (*p* = 0.046; *ηp*^2^ = 0.13), and when follow-ups were included (*p* = 0.048; *ηp*^2^ = 0.13).	
		Pain interference, PII, based on interference subscale of MPI and pain interference items of BPI	Pain interference significantly decreased over time in ACT (*p* = 0.016; *ηp*^2^ = 0.28) and TAU (*p* = 001; *ηp*^2^ = 0.29). Between-group differences were significant post-intervention, with significantly greater reductions in ACT (*p* = 0.024; *ηp*^2^ = 0.16), but not when including follow-ups (*p* = 0.107; *ηp*^2^ = 0.09).	
		Pain-related discomfort, five questions each rated using VAS scale	Pain-related discomfort significantly decreased over time in ACT (*p* = 0.003; *ηp*^2^ = 0.42) and TAU (*p* = 0.006; *ηp*^2^ = 0.32). Between-group differences were significant post-intervention, with significantly greater reductions in ACT (*p* = 0.001; *ηp*^2^ = 0.34), and when follow-ups were included (*p* = 0.031; *ηp*^2^ = 0.15).	
		Physical quality of life, subscale of SF-36	Physical quality of life significantly increased over time in ACT (*p* = 0.010; *ηp*^2^ = 0.31) and TAU (*p* = 0.004; *ηp*^2^ = 0.32). Between-group differences were not significant post-intervention (*p* = 0.367; *ηp*^2^ = 0.03) or when including follow-up (*p* = 0.224; *ηp*^2^ = 0.05).	
			Note: The groups were not fully comparable at follow-up, as TAU received a substantially greater amount of treatment after post-intervention assessments.	
Balter et al. (2021) [58] (Non-RCT without control group)	Pre-intervention Post-intervention	Depression, CES-DC	Depression significantly decreased pre–post-intervention (*p* < 0.001).	CYP higher in autistics traits showed significantly greater improvements in insomnia (*p* < 0.05) and emotional functioning (*p* < 0.05), but not pain interference (*p* < 0.07). The significant time–autism interactions for insomnia (*p* = 0.004) and emotional functioning (*p* = 0.007) remained significant when adjusting for age and gender. The non-significant time–pain interference (*p* = 0.057) interaction remained unchanged when adjusting for age and gender. No significant time–autism interactions were found for other outcomes (depression, pain intensity, physical functioning, psychological inflexibility, school functioning or social functioning), and no significant time–ADHD interactions were found. When CYP were split into above and below clinically significant autism or ADHD scores, between-group analyses showed that those with clinically significant levels of autistic traits and/or ADHD symptoms improved more for insomnia (*p* = 0.016) and emotional functioning *p* = 0.007) relative to those without clinically significant levels. Improvements in pain interference did not significantly differ by group (*p* = 0.293). Note: Autistic traits were measured using SRS, and ADHD symptoms were measured using Conners-3 ADHD Index (i.e., Conners Hyperactivity Index), Conners-3-P.
		Emotional functioning, subscale of PedsQL	Emotional functioning significantly increased pre–post-intervention (*p* = 0.010).	
		Insomnia, ISI	Insomnia did not significantly change pre–post-intervention. *	
		Pain intensity, item of the LPQ	Pain intensity significantly decreased pre–post-intervention (*p* = 0.016).	
		Pain interference, PII	Pain interference significantly decreased pre–post-intervention (*p* < 0.001).	
		Physical functioning, subscale of PedsQL	Physical functioning significantly increased pre–post-intervention (*p* = 0.001).	
		Psychological inflexibility, PIPS	Psychological inflexibility significantly decreased pre–post-intervention (*p* < 0.001)	
		School functioning, subscale of PedsQL	School functioning did not significantly change pre–post-intervention. *	
		Social functioning, subscale of PedsQL	Social functioning significantly increased pre–post-intervention (*p* = 0.019).	
			Note: Effect sizes could not be calculated, as *SD*s were not reported.	
Kemani et al. (2018) [59] (Non-RCT without control group)	Pre-intervention Post-intervention Three-month follow-up	Acceptance of pain, CPAQ-A	Acceptance significantly increased from pre-intervention to three-month follow-up (*p* < 0.001, *d* = 0.73).	Changes in parent psychological flexibility over time (pre, post and follow-up), measured using the PPFQ, were significantly (positively) associated with greater changes in adolescent pain acceptance while controlling for changes in all domains of functioning (*p* ≤ 0.01).
		Depression, subscale of BAPQ	Depression significantly decreased from pre-intervention to three-month follow-up (*p* < 0.001, *d* = 0.26).	
		Development, subscale of BAPQ	Development showed no significant main linear effect of time (*p* > 0.05, *d* = 0.10).	
		Family functioning, subscale of BAPQ	Family functioning scores significantly decreased (indicating higher levels) from pre-intervention to three-month follow-up (*p* < 0.001, *d* = 0.64).	
		General anxiety, subscale of BAPQ	General anxiety significantly decreased from pre-intervention to three-month follow-up (*p* < 0.001, *d* = 0.68).	
		Pain intensity (rated 0–10)	Pain intensity significantly decreased from pre-intervention to three-month follow-up (*p* = 0.020, *d* = 0.68).	
		Pain-specific anxiety, subscale of BAPQ	Pain-specific anxiety significantly decreased from pre-intervention to three-month follow-up (*p* = 0.020, *d* = 0.24).	
		Physical functioning, subscale of BAPQ	Physical functioning scores significantly decreased (indicating higher levels) from pre-intervention to three-month follow-up (*p* < 0.001, *d* = 0.42).	
		Social functioning, subscale of BAPQ	Social functioning scores significantly decreased (indicating higher levels) from pre-intervention to three-month follow-up (*p* < 0.001, *d* = 0.40).	
Vuorimaa et al. (2019) [60] (Non-RCT without control group)	Pre-intervention Six-month follow-up (from, pre-intervention) 12-month follow-up (from pre-intervention)	Depression, CDI	Note: No overall comparisons across time were conducted for the full sample, as participants were divided into two groups (based on pain risk profiles) for analyses. Nineteen adolescents were high-risk (total PPST score ≥ 3; psychosocial subscale score ≥ 3), and 13 adolescents were medium-risk (total PPST score ≥ 3; psychosocial subscale score 0 to 2). The potential moderating effects of PSST on outcomes were assessed (see right column).	For depression symptoms, there was a significant time–group interaction (*p* = 0.020). The high-risk group significantly decreased by 36% (*p* = 0.005). There was no significant change in the medium-risk group *.
		Pain catastrophising, subscale of PCQ		For pain catastrophising, the time–group interaction was significant (*p* < 0.001). The high-risk group significantly decreased by 19% (*p* = 0.008). There was no significant change in the medium-risk group *.
		Pain frequency, SPQ		For pain frequency, the significant time–group interaction was significant (*p* = 0.019). The high-risk group significantly improved by 25% (*p* = 0.005). There was no significant change in the medium-risk group *.
				At one-year follow-up of the 24 adolescents who remained, seven (37.5%, *p* = 0.008) initially in the high-risk group were reclassified as medium risk. No medium-risk adolescents were reclassified.
Zetterqvist et al. (2020), secondary analysis (Gentili et al., 2024) [61,64] (Non-RCT without control group)	Pre-intervention Post-intervention Four-month follow-up (completed at 17–25 weeks)	Depression, CEDS-DC	Depression significantly decreased from pre-intervention to follow-up (*p* < 0.001, *d* = 0.78).	Higher mean acceptance in the early treatment phase was related to a larger decrease in pain interference over the course of treatment (*p* = 0.049, *ρ* = −0.45 †), explaining 20% of the variance in outcome. Defusion (*p* = 0.795), values formulation (*p* = 0.607) and committed action (*p* = 0.541) showed no significant influence on pain interference. Note: ADPM, revised for the current study, was used to measure acceptance, defusion, values formation and committed action.
		Insomnia, ISI	Insomnia significantly decreased from pre-intervention to follow-up (*p* = 0.007, *d* = 0.54).	
		Pain interference, PII	Pain interference significantly decreased from pre-intervention to follow-up (*p* < 0.001, *d* = 1.09).	
		Psychological inflexibility, PIPS	Psychological inflexibility significantly decreased from pre-intervention to follow-up (*p* < 0.001, *d* = 1.13).	
		School functioning, subscale of PedsQL	School functioning from pre-intervention to follow-up did not significantly change (*p* = 0.885, *d* = 0.03).	

Note. ACT = acceptance and commitment therapy. CYP = children and young people. RCT = randomised controlled trial. TAU = treatment-as-usual. VAS = visual analogue scale. Studies are ordered by design (RCT to non-RCT without a control group). * = no *p*-value reported. † = report used *r* symbol instead of *ρ*. Measures used: ADPM = Acceptance and Defusion Process Measure [66]; BAPQ = Bath Adolescent Pain Questionnaire [67]; BPI = Brief Pain Inventory [68]; CDI = Children Depression Inventory, Finnish version [69,70]; CEDS-DC = Center for Epidemiological Studies Depression Scale for Children [71,72]; Conners-3-P = Conners 3rd Edition-Parent [73]; CPAQ-A = Chronic Pain Acceptance Questionnaire Adolescent [74]; FDI = Functional Disability Inventory—child form [75]; ISI = Insomnia Severity Index [76,77]; LPQ = Lubeck Pain-Screening Questionnaire [78]; MPI = Multidisciplinary Pain Inventory [79]; PAIRS = Pain and Impairment Relationship Scale [80]; PCQ = Pain Coping Questionnaire [81]; PedsQL = Pediatric Quality of Life Inventory [82,83]; PII = Pain Interference Index [47]; PIPS = Psychological Inflexibility in Pain Scale [84]; PPST = Pediatric Pain Screening Tool [85]; PRS = Pain Reactivity Scale [47]; SES = Self-Efficacy Scale [86]; SF-36 = Short Form-36 Health Survey [87]; SPQ = Structured Pain Questionnaire [88]; SRS = Social Responsiveness Scale [89]; TSK = Tampa Scale of Kinesiophobia [90].

**Table 5:** Summary of findings for diabetes.

Study (Design)	Time Points	CYP-Reported Psychosocial Outcomes	Findings on Intervention Effects on CYP-Reported Psychosocial Outcomes	Factors Associated with ACT Intervention Effects on CYP-Reported Psychosocial Outcomes
Alho et al. (2022) [48], secondary analysis (Alho et al., 2024) [56] (RCT)	Pre-intervention Post-intervention	Acceptance and mindfulness, CAMM	Acceptance and mindfulness change pre–post-intervention was not significantly different between groups (*p* = 0.218, *d* = 0.25).	Changes in diabetes-related psychological flexibility did not mediate the effect of ACT on anxiety (95% CI = −0.01 to 0.02).
		Anxiety, RBDI	Anxiety change pre–post-intervention was significantly different between groups (*p* = 0.012; d = 0.48). Change over time was significantly greater in ACT than TAU, with those in ACT showing a decrease in anxiety (*d* = 0.25).	
		Depression, RBDI	Depression change pre–post-intervention was not significantly different between groups (*p* = 0.814, *d* = 0.04).	
		Diabetes-related psychological flexibility, DAAS	Diabetes-related psychological flexibility change pre–post-intervention was significantly different between groups (*p* = 0.040; *d* = 0.29). Change over time was significantly greater in ACT than TAU, with those in ACT showing an increase in diabetes-related psychological flexibility (*d* = 0.26).	
		Quality of life–diabetes, KINDL^R^	Diabetes-related quality of life change pre–post-intervention was not significantly different between groups *(p* = 0.733, *d* = 0.05).	
		Quality of life–general, KINDL^R^	Diabetes-related general quality of life change pre–post-intervention was not significantly different between groups (*p* = 0.107, *d* = 0.29).	
Ataie Moghanloo et al. (2015) [50] (RCT)	Pre-intervention Post-intervention	Depression, RCDS	Depression was significantly lower in ACT than NI at post-intervention, after controlling the pre-test (*p* = < 0.001; *ηp*^2^ = 0.86 ^), with the means indicating depression decreased only in ACT.	Not studied.
		Feeling of guilt, EFGS	Feeling of guilt was significantly lower in ACT than NI at post-intervention, after controlling the pre-test (*p* = < 0.001; *ηp*^2^ = 0.85 ^), with the means indicating a greater decrease in ACT than NI.	
		Psychological wellbeing, SWLS	Psychological wellbeing was significantly higher in ACT than NI at post-intervention, after controlling the pre-test (*p* = < 0.001; *ηp*^2^ = 0.78 ^), with the means indicating a greater decrease in ACT than NI.	
Moazzezi et al. (2015) [49] (RCT)	Pre-intervention Post-intervention	Negative perceived stress, subscale of PSS	Negative perceived stress was significantly lower in ACT than NI at post-intervention, after controlling the pre-test (*p* = < 0.001; *ηp*^2^ = 0.92), with the means indicating a greater decrease in ACT than NI.	Not studied.
		Positive perceived stress, subscale of PSS	Positive perceived stress was significantly higher in ACT than NI at post-intervention, after controlling the pre-test (*p* = < 0.001; *ηp*^2^ = 0.85), with the means indicating a greater increase in ACT than NI.	
		Total perceived stress, PSS	Total perceived stress was significantly lower in ACT than NI at post-intervention, after controlling the pre-test (*p* = < 0.001; *ηp*^2^ = 0.20 ^), with the means indicating total perceived stress decreased only in ACT.	
		Special health self-efficacy, SHSES	Special health self-efficacy was significantly higher in ACT than NI at post-intervention, after controlling the pre-test (*p* = < 0.001; *ηp*^2^ = 0.85), with the means indicating special health self-efficacy increased only in ACT.	
Stefanescu et al. (2024) [62] (Non-RCT without a control group)	Pre-intervention Post-intervention	Acceptance, AADQ	Diabetes acceptance scores significantly decreased (indicating higher levels) pre–post-intervention (*p* < 0.001, *r* = 0.98).	Not studied.
		Patient–doctor relationship, PDRQ-9	Patient–doctor relationship significantly increased pre–post-intervention (*p* < 0.001, *r* = −0.86).	
		Psychological flexibility, CPFQ	Psychological flexibility significantly increased pre–post-intervention (*p* < 0.001, *d* = 0.54).	
		Stress, PSS-C	Stress significantly decreased pre–post-intervention (*p* < 0.001, *d* = 1.18).	

Note. ACT = acceptance and commitment therapy. CYP = children and young people. NI = no intervention. RCT = randomised controlled trial. TAU = treatment as usual. Studies are ordered by design (RCT to non-RCT without a control group). ^ = effect size calculated. Measures used: AADQ = Acceptance and Action Diabetes [91]; CAMM = Child and Adolescent Mindfulness Measure [92]; CPFQ = Children’s Psychological Flexibility Questionnaire [93]; DAAS = Diabetes Acceptance and Action Scale [94]; EFGS = Eysenck Feeling of Guilt Scale, Iranian version [95]; KINDL^R^ Revised Children’s Quality of Life Questionnaire [92]; PDRQ-9 = Patient–Doctor Relationship Questionnaire [96]; PSS = Perceived Stress Scale [97]; PSS-C = Perceived Stress Scale for Children [98]; RBDI = Revised Beck Depression Inventory [99]; RCDS = Reynolds Child Depression Scale [100]; SHSES = Special Health Self-Efficacy Scale [101]; SWLS = Satisfaction with Life Scale [102].

**Table 7:** Summary of findings for cancer.

Study (Design)	Time Points	CYP-Reported Psychosocial Outcomes	Findings on Intervention Effects on CYP-Reported Psychosocial Outcomes
Asadi et al. (2016) [53] (RCT)	Pre-intervention Post-intervention One-month follow-up	Anger, ChIA	For anger, a significant time–group interaction effect was reported (*p* < 0.001, *ƞ*^2^ = 0.87 ^). Change over time was significantly greater in ACT than meeting without offering a solution, with means indicating anger decreased in ACT.
Ebrahimi et al. (2022) [57] (Non-RCT with control group)	Pre-intervention Post-intervention	Resilience, CD-RISC Self-concept, PHCSCS	Resilience was significantly higher in ACT than NI post-intervention after removing the interaction effect (*p* = 0.002, *ηp*^2^ = 0.48 ^). Self-concept was significantly higher in ACT than NI post-intervention after removing the interaction effect (*p* = 0.001, *ηp*^2^ = 0.31 ^).

Note. ACT = acceptance and commitment therapy. CYP = children and young people. NI = no intervention. RCT = randomised controlled trial. Studies are ordered by design (RCT to non-RCT). ^ = effect size calculated. Measures used: CD-RISC = Connor–Davidson Resilience Scale [115]; ChIA = Children’s Inventory of Anger [116]; PHCSCS = Piers–Harris Children’s Self-Concept Scale [117].

## Data Availability

No new data were created or analysed in this study. The data supporting the findings of this systematic review are within the article.

## Supplementary Materials

The following supporting information can be downloaded at https://www.mdpi.com/article/10.3390/children13050672/s1, S1: Preferred Reporting Items for Systematic review and Meta-Analysis (PRISMA) 2020 Checklist [37] with Synthesis Without Meta-Analysis (SWiM) 2019 Reporting Items [41]; S2: Search Strategy; S3: Modified Cochrane Data Collection Form for Intervention Reviews [40]; S4: Quality Appraisal per Domain Using the The Cochrane Risk of Bias 2 (RoB 2) Tool [43] and The Cochrane Risk of Bias in Non-Randomised Studies of Interventions (ROBINS-I) Tool [44].

## Abbreviations

The following abbreviations are used in this manuscript:

ACT	Acceptance and commitment therapy
CBT	Cognitive behavioural therapy
CYP	Children and young people
LTC	Long-term physical health condition
NI	No intervention
RCT	Randomised controlled trial
PICO	Patient, Intervention, Comparison and Outcome
PRISMA	Preferred Reporting Items for Systematic Reviews and Meta-Analysis
PROSPERO	Prospective Register of Systematic Reviews
RoB 2	Risk of Bias 2
ROBINS-I	Risk of Bias in Non-Randomised Studies of Interventions
SWiM	Synthesis without Meta-Analysis
TAU	Treatment as usual
