# Supplementary material for: Acceptance and Commitment Therapy for Psychosocial Outcomes in Children and Young People with Long-Term Physical Health Conditions: Systematic Review of Intervention Studies
Source: Children (Basel). 2026 May 12;13(5):672. doi: 10.3390/children13050672 (PMC13205000; doi:10.3390/children13050672)
Supplement: Supplementary file 1 [file children-13-00672-s001.zip › S1.pdf]

S1

Preferred Reporting Items for Systematic review and Meta-Analysis (PRISMA) 2020 Checklist [37] with Synthesis Without Meta-Analysis (SWiM) 2019 Reporting Items [41]

Section and Topic	Item #	Checklist item	Location where item is reported
**TITLE**			
Title	1	Identify the report as a systematic review.	Page 1.
**ABSTRACT**			
Abstract	2	See the PRISMA 2020 for Abstracts checklist.	Pages 1-2.
**INTRODUCTION**			
Rationale	3	Describe the rationale for the review in the context of existing knowledge.	Pages 2-3.
Objectives	4	Provide an explicit statement of the objective(s) or question(s) the review addresses.	Page 3.
**METHODS**			
Eligibility criteria	5	Specify the inclusion and exclusion criteria for the review and how studies were grouped for the syntheses.	Pages 4-5 (including Table 1).
Information sources	6	Specify all databases, registers, websites, organisations, reference lists and other sources searched or consulted to identify studies. Specify the date when each source was last searched or consulted.	Page 4.
Search strategy	7	Present the full search strategies for all databases, registers and websites, including any filters and limits used.	Page 4 and S2.
Selection process	8	Specify the methods used to decide whether a study met the inclusion criteria of the review, including how many reviewers screened each record and each report retrieved, whether they worked independently, and if applicable, details of automation tools used in the process.	Page 5.
Data collection process	9	Specify the methods used to collect data from reports, including how many reviewers collected data from each report, whether they worked independently, any processes for	Page 5 and S3.

Section and Topic	Item #	Checklist item	Location where item is reported
		obtaining or confirming data from study investigators, and if applicable, details of automation tools used in the process.	
Data items	10a	List and define all outcomes for which data were sought. Specify whether all results that were compatible with each outcome domain in each study were sought (e.g. for all measures, time points, analyses), and if not, the methods used to decide which results to collect.	Page 5 and S3.
	10b	List and define all other variables for which data were sought (e.g. participant and intervention characteristics, funding sources). Describe any assumptions made about any missing or unclear information.	Page 5 and S3.
Study risk of bias assessment	11	Specify the methods used to assess risk of bias in the included studies, including details of the tool(s) used, how many reviewers assessed each study and whether they worked independently, and if applicable, details of automation tools used in the process.	Pages 5-6.
Effect measures	12	Specify for each outcome the effect measure(s) (e.g. risk ratio, mean difference) used in the synthesis or presentation of results.	Page 5.
Synthesis methods	13a	Describe the processes used to decide which studies were eligible for each synthesis (e.g. tabulating the study intervention characteristics and comparing against the planned groups for each synthesis (item #5)).	Page 5.
	13b	Describe any methods required to prepare the data for presentation or synthesis, such as handling of missing summary statistics, or data conversions.	Page 5.
	13c	Describe any methods used to tabulate or visually display results of individual studies and syntheses.	Page 5.
	13d	Describe any methods used to synthesize results and provide a rationale for the choice(s). If meta-analysis was performed, describe the model(s), method(s) to identify the presence and extent of statistical heterogeneity, and software package(s) used.	Page 5.
	13e	Describe any methods used to explore possible causes of heterogeneity among study results (e.g. subgroup analysis, meta-regression).	Page 5.
	13f	Describe any sensitivity analyses conducted to assess robustness of the synthesized results.	N/A - see item 15.
Reporting bias	14	Describe any methods used to assess risk of bias due to missing results in a synthesis (arising	Pages 5-6.

Section and Topic	Item #	Checklist item	Location where item is reported
assessment		from reporting biases).	
Certainty assessment	15	Describe any methods used to assess certainty (or confidence) in the body of evidence for an outcome.	N/A - not formally conducted, due to substantial clinical and methodological heterogeneity and large number of outcomes across different long-term physical health conditions. Thus, certainty of evidence grading was unlikely to be meaningful, in line with SWiM guidance which recognises this assessment may not always be suitable/applicable.
**RESULTS**			
Study selection	16a	Describe the results of the search and selection process, from the number of records identified in the search to the number of studies included in the review, ideally using a flow diagram.	Page 6 (including Figure 1).
	16b	Cite studies that might appear to meet the inclusion criteria, but which were excluded, and explain why they were excluded.	Figure 1.
Study characteristics	17	Cite each included study and present its characteristics.	Pages 6-14 (including Tables 2 and 3).
Risk of bias in studies	18	Present assessments of risk of bias for each included study.	Pages 15-16 (including Figure 2).
Results of individual studies	19	For all outcomes, present, for each study: (a) summary statistics for each group (where appropriate) and (b) an effect estimate and its precision (e.g. confidence/credible interval), ideally using structured tables or plots.	Pages 16-28 (including Table 4-8).

Section and Topic	Item #	Checklist item	Location where item is reported
Results of syntheses	20a	For each synthesis, briefly summarise the characteristics and risk of bias among contributing studies.	Pages 16-28 (including Table 4-8).
	20b	Present results of all statistical syntheses conducted. If meta-analysis was done, present for each the summary estimate and its precision (e.g. confidence/credible interval) and measures of statistical heterogeneity. If comparing groups, describe the direction of the effect.	Pages 16-28 (including Table 4-8).
	20c	Present results of all investigations of possible causes of heterogeneity among study results.	Pages 16-28 (including Table 4-8).
	20d	Present results of all sensitivity analyses conducted to assess the robustness of the synthesized results.	N/A - see item 15.
Reporting biases	21	Present assessments of risk of bias due to missing results (arising from reporting biases) for each synthesis assessed.	Pages 15-16 (including Figure 2).
Certainty of evidence	22	Present assessments of certainty (or confidence) in the body of evidence for each outcome assessed.	N/A - see item 15.
**DISCUSSION**			
Discussion	23a	Provide a general interpretation of the results in the context of other evidence.	Pages 28-29.
	23b	Discuss any limitations of the evidence included in the review.	Page 30.
	23c	Discuss any limitations of the review processes used.	Page 30.
	23d	Discuss implications of the results for practice, policy, and future research.	Pages 30-31.
**OTHER INFORMATION**			
Registration and protocol	24a	Provide registration information for the review, including register name and registration number, or state that the review was not registered.	Pages 2 and 4.
	24b	Indicate where the review protocol can be accessed, or state that a protocol was not prepared.	Pages 2 and 4.
	24c	Describe and explain any amendments to information provided at registration or in the protocol.	Page 5.
Support	25	Describe sources of financial or non-financial support for the review, and the role of the funders or sponsors in the review.	Page 32.

Section and Topic	Item #	Checklist item	Location where item is reported
Competing interests	26	Declare any competing interests of review authors.	Page 32.
Availability of data, code and other materials	27	Report which of the following are publicly available and where they can be found: template data collection forms; data extracted from included studies; data used for all analyses; analytic code; any other materials used in the review.	Template data collection form(s). Data extracted from included studies presented in systematic review.

SWiM is intended to complement and be used as an extension to PRISMA		
**SWiM reporting item**	**Item description**	**Page in manuscript where item is reported**
**1** Grouping studies for synthesis	1a) Provide a description of, and rationale for, the groups used in the synthesis (e.g., groupings of populations, interventions, outcomes, study design)	Page 5.
	1b) Detail and provide rationale for any changes made subsequent to the protocol in the groups used in the synthesis	N/A - no changes made to grouping(s).
**2** Describe the standardised metric and transformation methods used	Describe the standardised metric for each outcome. Explain why the metric(s) was chosen, and describe any methods used to transform the intervention effects, as reported in the study, to the standardised metric, citing any methodological guidance consulted	Page 5.
**3** Describe the synthesis methods	Describe and justify the methods used to synthesise the effects for each outcome when it was not possible to undertake a meta-analysis of effect estimates	Page 5.
**4** Criteria used to prioritise results for summary and synthesis	Where applicable, provide the criteria used, with supporting justification, to select the particular studies, or a particular study, for the main synthesis or to draw conclusions from the synthesis (e.g., based on study design, risk of bias assessments, directness in relation to the review question)	Page 5.

SWiM is intended to complement and be used as an extension to PRISMA		
**SWiM reporting item**	**Item description**	**Page in manuscript where item is reported**
**5** Investigation of heterogeneity in reported effects	State the method(s) used to examine heterogeneity in reported effects when it was not possible to undertake a meta-analysis of effect estimates and its extensions to investigate heterogeneity	Page 5.
**6** Certainty of evidence	Describe the methods used to assess certainty of the synthesis findings	N/A - not formally conducted, due to substantial clinical and methodological heterogeneity and large number of outcomes across different long-term physical health conditions. Thus, certainty of evidence grading was unlikely to be meaningful, in line with SWiM guidance which recognises this assessment may not always be suitable/applicable.
**7** Data presentation methods	Describe the graphical and tabular methods used to present the effects (e.g., tables, forest plots, harvest plots).<br>Specify key study characteristics (e.g., study design, risk of bias) used to order the studies, in the text and any tables or graphs, clearly referencing the studies included	Page 5.
**8** Reporting results	For each comparison and outcome, provide a description of the synthesised findings, and the certainty of the findings. Describe the result in language that is consistent with the question the synthesis addresses, and indicate which studies contribute to the synthesis	Pages 16-28 (including Table 4-8).
Discussion		

SWiM is intended to complement and be used as an extension to PRISMA		
**SWiM reporting item**	**Item description**	**Page in manuscript where item is reported**
**9** Limitations of the synthesis	Report the limitations of the synthesis methods used and/or the groupings used in the synthesis, and how these affect the conclusions that can be drawn in relation to the original review question	Page 30.
