# Supplementary material for: Acceptance and Commitment Therapy for Psychosocial Outcomes in Children and Young People with Long-Term Physical Health Conditions: Systematic Review of Intervention Studies
Source: Children (Basel). 2026 May 12;13(5):672. doi: 10.3390/children13050672 (PMC13205000; doi:10.3390/children13050672)
Supplement: Supplementary file 1 [file children-13-00672-s001.zip › S2.pdf]

S2

Search Strategy

CINAHL (EBSCO)

1	TI (Child* OR Adolescen* OR Teen* OR youth* OR young* OR juvenile* OR paediatric* OR pediatric* OR Boy* OR Girl* OR Schoolchild* OR Minor* OR "Under 18")
2	AB (Child* OR Adolescen* OR Teen* OR youth* OR young* OR juvenile* OR paediatric* OR pediatric* OR Boy* OR Girl* OR Schoolchild* OR Minor* OR "Under 18")
3	1 or 2
4	TI ("acceptance and commitment therapy" OR "DNA-V" OR "Discoverer Noticer Advisor?Values" OR "psychological flexibility")
5	AB ("acceptance and commitment therapy" OR "DNA-V" OR "Discoverer Noticer Advisor?Values" OR "psychological flexibility")
6	4 or 5
7	3 and 6

Cochrane Library

1	Title Abstract Keyword (Child* or Adolescen* or Teen* or youth* or young* or juvenile* or paediatric* or pediatric* or Boy* or Girl* or Schoolchild* or Minor* or "Under 18")
2	Title Abstract Keyword ("acceptance and commitment therapy" or "DNA-V" or "Discoverer Noticer Advisor?Values" or "psychological flexibility")
3	1 and 2

Embase (Ovid)

1	(Child* or Adolescen* or Teen* or youth* or young* or juvenile* or paediatric* or pediatric* or Boy* or Girl* or Schoolchild* or Minor* or "Under 18").ti,ab,kf.
2	("acceptance and commitment therapy" or "DNA-V" or "Discoverer Noticer Advisor?Values" or "psychological flexibility").ti,ab,kf.
3	1 and 2

MEDLINE (Ovid)

1	(Child* or Adolescen* or Teen* or youth* or young* or juvenile* or paediatric* or pediatric* or Boy* or Girl* or Schoolchild* or Minor* or "Under 18").ti,ab,kf.

2 ("acceptance and commitment therapy" or "DNA-V" or "Discoverer Noticer Advisor?Values" or "psychological flexibility").ti,ab,kf.
3 1 and 2

PsycInfo (Ovid)

1 (Child* or Adolescen* or Teen* or youth* or young* or juvenile* or paediatric* or pediatric* or Boy* or Girl* or Schoolchild* or Minor* or "Under 18").ti,ab,kf.
2 ("acceptance and commitment therapy" or "DNA-V" or "Discoverer Noticer Advisor?Values" or "psychological flexibility").ti,ab,kf.
3 1 and 2
