# Supplementary material for: Acceptance and Commitment Therapy for Psychosocial Outcomes in Children and Young People with Long-Term Physical Health Conditions: Systematic Review of Intervention Studies
Source: Children (Basel). 2026 May 12;13(5):672. doi: 10.3390/children13050672 (PMC13205000; doi:10.3390/children13050672)
Supplement: Supplementary file 1 [file children-13-00672-s001.zip › S3.pdf]

S3

Data Extraction Form [40]

THE COCHRANE COLLABORATION®

Data collection form

Intervention review – RCTs and non-RCTs

This form can be used as a guide for developing your own data extraction form. Sections can be expanded and added, and irrelevant sections can be removed. It is difficult to design a single form that meets the needs of all reviews, so it is important to consider carefully the information you need to collect, and design your form accordingly. Information included on this form should be comprehensive, and may be used in the text of your review, 'Characteristics of included studies' table, risk of bias assessment, and statistical analysis.

Notes on using data extraction form:

- Be consistent in the order and style you use to describe the information for each report.
- Record any missing information as unclear or not described, to make it clear that the information was not found in the study report(s), not that you forgot to extract it.
- Include any instructions and decision rules on the data collection form, or in an accompanying document. It is important to practice using the form and give training to any other authors using the form.

Modified for use by Rachel Batchelor for *Acceptance and Commitment Therapy for Psychosocial Outcomes in Children and Young People with Long-Term Conditions: Systematic Review of Intervention Studies.*

1. General Information

Date form completed *(dd/mm/yyyy)*	
Name of person extracting data	
Reference citation	
Report title	
Report authors	
Report author contact details	
Publication type	
Study funding sources *(including role of funders)*	
Possible conflicts of interest *(for study authors)*	
Notes:	

2. Characteristics of included studies

2.1. Methods

	Descriptions as stated in report/paper		Location in text or source
Aim(s) of study *(primary and secondary)*			
Design *(e.g. RCT, non-RCT)*			
Follow-up time points *(yes – specify how many and when – or no)*			
Start date			
End date			
Duration of participation *(from recruitment to last follow-up)*			
Ethical approval needed/ obtained for study	☐ Yes ☐ No ☐ Unclear		
Notes:			

2.2. Participants (Characteristics of Children and Young People)

	Description *Include comparative information for each intervention or comparison group if available*		Location in text or source
Population description *(from which study participants are drawn)*			
Long-term physical health condition(s)			
Country			
Setting *(including location and social context)*			
Inclusion criteria			
Exclusion criteria			
Method of recruitment of participants *(e.g. phone, mail, clinic patients)*			
Informed consent obtained	☐ Yes ☐ No ☐ Unclear		

Total number randomised *(or total number at start for non-RCTs)*		
Clusters *(if applicable, number, type, number people per cluster)*		
Baseline imbalances *(e.g. age, gender, size of groups)*		
Withdrawals and exclusions *(if not provided below by outcome)*		
Age of children and young people *(mean, standard deviation, range)*		
Gender of children and young people		
Race/Ethnicity		
Other relevant sociodemographics		
Subgroups measure		
Subgroups reported		
Notes:		

2.3. Intervention groups

Intervention Group 1 copy and paste for other intervention groups if necessary

	Description as stated in report/paper	Location in text or source
Name of intervention		
Method of group allocation *(e.g. randomised, naturalistic)*		
Number of participants in group		
Theoretical basis of intervention *(include key references)*		
Description of contents *(e.g. core processes of psychological flexibility covered, parental involvement)*		
Delivery structure and dose *(e.g. duration of intervention, frequency of sessions, number of sessions, length of sessions)*		
Delivery format *(e.g. individual, group)*		
Delivery method *(e.g. face-to-face, online)*		
Providers/facilitators *(e.g. profession, training, ethnicity etc. if relevant)*		
Co-interventions		
Fidelity to intervention *(is each component of intervention being delivered in a comparable manner to all study participants over time?)*		
Compliance *(number of people who dropped out and at what stage)*		
Adverse events		
Notes:		

Control Group 1 copy and paste for other control groups if necessary

	Description as stated in report/paper	Location in text or source

Type of control group *(e.g. active intervention [specify what], treatment-as-usual [specify what], no intervention, waiting list, other - specify)*		
Method of group allocation *(e.g. randomised, naturalistic)*		
Number of participants in group		
Theoretical basis of control intervention *(if relevant)*		
Description of control intervention *(if relevant, include sufficient detail for replication including contents, delivery structure and dose, delivery format, delivery method and provides/facilitators)*		
Notes:		

2.4. Outcomes

Note: Only extract psychosocial outcomes completed by children and young people (self-reported)

Copy and paste table for each outcome.

Psychosocial outcome

	Description as stated in report/paper		Location in text or source
Outcome name			
Time points measured *(specify whether from start or end of intervention)*			
Time points reported			
Outcome definition *(with diagnostic criteria if relevant)*			
Person measuring/ reporting			
Unit of measurement *(if relevant)*			
Scales: upper and lower limits *(indicate whether high or low score is good)*			
Is outcome/tool validated?	☐ Yes ☐ No ☐ Unclear		
Imputation of missing data *(e.g. assumptions made for ITT analysis)*			
Notes:			

3. Data and analysis

Copy and paste the appropriate table for each outcome, including additional tables for each time point and subgroup as required.

Psychosocial outcome

<table>
<tr><td></td><td colspan="6">Description as stated in report/paper</td><td>Location in text or source</td></tr>
<tr><td>Comparison (groups being compared)</td><td colspan="6"></td><td></td></tr>
<tr><td>Outcome</td><td colspan="6"></td><td></td></tr>
<tr><td>Subgroups (if any)</td><td colspan="6"></td><td></td></tr>
<tr><td>Time point
(specify from start or end of intervention)</td><td colspan="6"></td><td></td></tr>
<tr><td rowspan="2">Results
Mean, SD, number of participants</td><td colspan="3">Intervention (also list other timepoints if needed)</td><td colspan="3">Comparison (also list other timepoints if needed)</td><td rowspan="2"></td></tr>
<tr><td>Pre</td><td>Post</td><td>Follow-up(s)</td><td>Pre</td><td>Post</td><td>Follow-up(s)</td></tr>
<tr><td>Differences between timepoints
- Pre-post
- Post-FU
- Pre-FU</td><td colspan="6"></td><td></td></tr>
<tr><td>Any other results reported
- Odds ratio
- Assumed risk estimate
- Risk difference
- p-value
- CI
- Power and sample size calculations
- Level of power achieved</td><td colspan="6"></td><td></td></tr>
<tr><td>Effect size(s) (reported or calculate, specify if calculated)</td><td colspan="6"></td><td></td></tr>
</table>

Missing data<br>- *Number of missing participants and reason*<br>- *How was missing data handled (intention to treat/imputation or exclusion?)*		
Statistical methods used and appropriateness of these *(e.g. adjustment for correlation)*		
Additional analysis *(on factors associated with effects on outcomes e.g. mediation, moderation, etc.)*		
Notes:		

Acceptability

	Description as stated in report/paper	Location in text or source
Measure of acceptability/satisfaction		
Results		
Notes:		

4. Other information

	Description as stated in report/paper	Location in text or source
Other outcomes included in the trial but not relevant for the review		
Economic information *(i.e. intervention cost, changes in other costs as result of intervention)*		
Key conclusions of study authors		
References to other relevant studies		
Correspondence required for further study information *(from whom, what and when)*		
Notes:		

.
