# Supplementary material for: Acceptance and Commitment Therapy for Psychosocial Outcomes in Children and Young People with Long-Term Physical Health Conditions: Systematic Review of Intervention Studies
Source: Children (Basel). 2026 May 12;13(5):672. doi: 10.3390/children13050672 (PMC13205000; doi:10.3390/children13050672)
Supplement: Supplementary file 1 [file children-13-00672-s001.zip › S4.pdf]

S4

Quality Appraisal per Domain Using the The Cochrane Risk of Bias 2 (RoB 2) Tool [43] and The Cochrane Risk of Bias in Non-Randomised Studies of Interventions (ROBINS-I) Tool [44]

The Cochrane Risk of Bias 2 (RoB 2) [43] tool was used to assess the quality of randomised controlled trials (RCTs). The Cochrane Risk of Bias in Non-Randomised Studies of Interventions (ROBINS-I) [44] tool was used to assess the quality of non-RCTs. Quality was assessed for each outcome of interest (i.e., CYP-reported psychosocial). As ratings were identical across outcomes within each study, a single set of combined outcome ratings have been presented per study. The effect of interest for the current systematic review was that of assignment to intervention. The quality appraisal for each domain has been summarised below.

RCTs

The overall risk of bias for most RCTs was rated as 'high' [46–52,54]. One study was rated as having 'some concerns' overall [53]. However, this rating should be interpreted with caution, as several domains had insufficient information and were therefore rated as 'some concerns' in accordance with the tool's guidance, which specifies that domain-level ratings cannot be 'no information' [43].

Randomisation

A strength for several studies was randomisation, as they reported appropriate methods of sequence generation (random and concealed allocation sequence) and did not indicate substantial baseline imbalance. Five studies did not report sufficient information about the randomisation process, rendering their rating as 'some concerns' [49–51,53,54].

Deviations from Interventions

An area of weakness for most studies was deviations from interventions. For all studies, participants and facilitators were aware of assigned interventions. No deviations from the intended intervention were indicated, although one study did not provide any information about the intervention content [53. All but two studies, one which indicated no dropouts [53] and one which used intention-to-treat analyses [47], did not include all randomised participants in the analyses; this meant they were rated as high for part two of, and consequently overall for, this domain.

Missing Outcome Data

Ratings for missing outcome data were mixed. Apart from one study which indicated no dropouts [53], dropout rates were high for most studies, varying between 7.14% and 37.5% from randomisation to final data collection point. Of the five studies that provided dropout reasons, two studies indicated non-health related reasons [51,54]. Of the remaining three studies, two studies indicated 5% or less of dropouts were due to health-related reasons [49,50]. The third study indicated that over 20% of dropouts were due to health-related reasons and dropout rates differed between groups [46]. In those that did not provide reasons, dropout rates were similar between groups [47,48,52].

Measurement of Outcome

Another area of weakness was measurement of outcome. While all studies had appropriate methods of outcome measurement, comparable between the groups, all outcomes were self-reported (as required for inclusion eligibility). This meant the outcome assessors were the participants and

therefore were aware of the intervention received and were potentially influenced by this. The control groups were TAU or NI for all but four studies [46,51,53,54], making it more likely that the outcome may have been influenced by the intervention received.

Selection of Results

Three studies [48,51,52] were pre-registered, yet did not have pre-specified analysis plan in such registrations. Therefore, for all studies, there was no information on whether the result being assessed is likely to have been selected, based on the results, from multiple eligible outcome measurements (e.g., scales, definitions, time points) within the outcome domain and from multiple eligible analyses of the data, therefore fitting with the criteria for some concerns.

Non-RCTs

The overall risk of bias for non-RCTs were rated as 'serious', indicating 'important problems' [44]. However, without the 'measurement of outcome' domain, which was rated as serious for all studies due to self-reported psychosocial measures being used (as required for inclusion eligibility), two studies would be rated as 'moderate' [58,59], indicating they provide sound evidence for a non-RCT but cannot be considered comparable to a well performed randomised trial [44].

Confounding

One of the greatest areas of weakness of the included non-RCTs was the potential for confounding. Only two studies controlled for potential confounders in the analyses [58,59]. The remaining studies were rated as serious rather than critical, as the exclusion criteria of all studies removed the risks of some confounders.

Selection of participants

Considering strengths of the studies, selection of participants into all studies were not based on participant characteristics observed after the start of the intervention. It was indicated that the start of the intervention coincided for most participants and the follow-up(s) coincided for most participants in all but one study. In one study [61], the four-month follow-up was completed by participants between 17-25 weeks post intervention, with 64% not completed within the two-week timeframe.

Identification Classification

For most studies, intervention groups were well defined. One study did not provide information about the intervention was not in English [57].

Deviations from Interventions

For most studies, no deviations from the intended intervention beyond what would be expected in usual practice were indicated. There was not sufficient information to assess this in one study, as information about the intervention was not in English [57].

Missing Outcome Data

A small number of studies [57,62] indicated no, or below 5%, dropouts or missing outcome data and were therefore rated as low for this domain. In the remaining studies, rates of dropout and missing data ranged from 14.3% to 42.8%. As none of these studies had control groups, determining

the similarity of proportions of and reasons for missing data across groups was not relevant. However, no studies provided dropout reasons, meaning there was insufficient evidence to rule out bias from missing outcome data, resulting in moderate ratings.

Measurement of Outcome

Although methods of outcome assessment were comparable across groups and systematic measurement errors related to intervention status were not indicated, all studies were rated as serious, as outcome measures were self-reported, and therefore subjective, and assessors were aware of the intervention received by participants (i.e., not blinded).

Selection of Results

Regarding the selection of the reported results, only one study [61], specified in a pre-registered protocol that follow-up data would be gathered at four months and one-year post-intervention. However, the four-month data was gathered 17-25 weeks post-intervention and the one-year follow-up data was not mentioned at all in the reports, indicating potential selective reporting. Although most other studies were internally consistent across the methods and results, they did not have pre-registered protocol or a-prioi statistical analysis plan, meaning there was insufficient information to ascertain whether selective reporting may have occurred).
